# Effects of extracorporeal carbon dioxide removal in facilitating ultra-protective ventilation strategies for patients with acute respiratory distress syndrome: a systematic review and meta-analysis

**DOI:** 10.3389/fmed.2025.1707596

**Published:** 2025-11-12

**Authors:** Weifeng Zhen, Xiaoning Zhang, Zhenhua Shi, Yu Zhu

**Affiliations:** Department of Intensive Care Unit, PLA 983rd Hospital, Tianjin, China

**Keywords:** extracorporeal carbon dioxide removal, ultra-protective ventilation, acute respiratory distress syndrome, systematic review, meta-analysis

## Abstract

**Background:**

Although low tidal volume ventilation has been shown to reduce mortality in patients with acute respiratory distress syndrome (ARDS), overall mortality remains high (30%−40%). Ultra-protective ventilation (≤4 mL/kg predicted body weight) has the potential to further decrease ventilator-induced lung injury but may result in severe hypercapnia. Extracorporeal carbon dioxide removal (ECCO_2_R) could facilitate ultra-protective ventilation by alleviating carbon dioxide retention; however, supporting evidence remains limited.

**Objective:**

To evaluate the efficacy of ECCO_2_R in enabling ultra-protective ventilation strategies in patients with ARDS.

**Methods:**

A systematic search was conducted in PubMed, Embase, Web of Science, and the Cochrane Library for studies published up to June 2025 that met predefined inclusion criteria. Primary outcomes included changes in gas exchange and ventilator settings 24 h after initiating ECCO_2_R. All analyses were performed using a random-effects model. Sensitivity and subgroup analyses were conducted to further explore the findings.

**Results:**

Fourteen studies involving 593 ARDS patients were included. ECCO_2_R significantly reduced driving pressure (weighted mean difference [WMD]: −3.70 cmH_2_O; 95% CI: −4.05 to −3.34; *P* < 0.001), plateau pressure (WMD: −3.26 cmH_2_O; 95% CI: −3.70 to −2.82; *P* < 0.001), and tidal volume (WMD: −1.68 mL/kg; 95% CI: −1.81 to −1.55; *P* < 0.001) at 24 h, while it increased positive end-expiratory pressure (WMD: 0.64 cmH_2_O; 95% CI: 0.44 to 0.85; *P* < 0.001). No significant changes were observed in PaO_2_/FiO_2_ ratio, pCO_2_, or pH (*P* > 0.05). The pooled 28-day mortality rate was 29% (95% CI: 19%−38%). Notable complications included bleeding (15%; 95% CI: 8%−21%), circuit clotting (19%; 95% CI: 13%−26%), and hemolysis (15%; 95% CI: 5%−25%).

**Conclusion:**

ECCO_2_R facilitates the implementation of ultra-protective ventilation by significantly improving respiratory mechanics and mitigating the hypercapnia that would otherwise result from ultra-low tidal volumes. However, its use is associated with a notable risk of device-related complications, necessitating careful patient selection and expert management.

**Systematic review registration:**

INPLASY platform (registration number: INPLASY202570067.

## Introduction

Acute respiratory distress syndrome (ARDS) is a critical condition commonly encountered in intensive care units. It is pathologically characterized by diffuse damage to the alveolar–capillary membrane, resulting in refractory hypoxemia and decreased lung compliance (1). Although low tidal volume ventilation has been shown to significantly reduce 28-day mortality, the overall mortality rate remains high, ranging from 30% to 40%, underscoring the need for more effective ventilation strategies (2). While standard low tidal volume ventilation mitigates ventilator-induced lung injury, it does not entirely eliminate the shear stress caused by repetitive alveolar opening and collapse (3). Pathophysiological studies have identified the “baby lung” phenomenon in ARDS, wherein conventional tidal volumes may lead to regional overdistension of the remaining functional lung units (4).

Recently, ultra-protective ventilation—defined as tidal volume ≤ 4 mL/kg predicted body weight—has attracted interest for its potential to further reduce lung injury (5). However, its application is constrained by the risk of severe hypercapnia and acid–base disturbances (6). Animal studies indicate that reducing tidal volumes below 4 mL/kg helps attenuate injury to the alveolar–epithelial–endothelial barrier; nevertheless, consequent carbon dioxide (CO_2_) retention may lead to pulmonary hypertension, increased cerebral blood flow, and myocardial suppression (7). Therefore, striking a balance between lung protection and CO_2_ clearance remains a major challenge in optimizing ventilation strategies for ARDS.

Extracorporeal CO_2_ removal (ECCO_2_R) utilizes hollow-fiber membrane gas exchange, either through extracorporeal membrane oxygenation (ECMO) or dedicated low-flow devices, to selectively remove CO_2_ from the blood (8). Recent clinical studies suggest that ECCO_2_R-assisted ventilation can reduce plateau pressure to below 25 cmH_2_O while maintaining arterial pH above 7.25 in patients with ARDS (9). However, most available studies are limited by small sample sizes, methodological heterogeneity, and a lack of control groups. Moreover, several key clinical uncertainties remain, including the optimal timing of intervention, standardization of technical parameters, and the effect on definitive patient outcomes such as mortality. In light of these gaps, this systematic review and meta-analysis aims to evaluate the impact of ECCO_2_R-facilitated ultra-protective ventilation on respiratory mechanics, ventilation parameters, and complications in patients with ARDS.

## Methods

### Data sources, search strategy, and selection criteria

This systematic review was conducted in accordance with the Preferred Reporting Items for Systematic Reviews and Meta-Analyses (PRISMA) guidelines (10). The study protocol was registered on the INPLASY platform (registration number: INPLASY202570067). A comprehensive literature search was performed in PubMed, Embase, Web of Science, and the Cochrane Library for studies published from database inception through June 2025. Search strategies incorporated both Medical Subject Headings (MeSH) and free-text terms, including: “extracorporeal carbon dioxide removal,” “ECCO2R,” “acute respiratory distress syndrome,” “ARDS,” “ultra-protective ventilation,” and “low tidal volume ventilation.” The complete search strategies for each database are detailed in Supplementary File 1. Manual searches of conference abstracts, gray literature, and backward citation tracking were also conducted to minimize the risk of study omission.

Two reviewers independently screened the identified literature based on a predefined protocol. Any discrepancies were resolved through team consensus. Studies were included if they met the following criteria: (1) population: patients diagnosed with ARDS according to the Berlin Definition; (2) intervention: ECCO_2_R-assisted ultra-protective ventilation, defined as tidal volume ≤ 4 mL/kg predicted body weight (PBW); (3) study designs: randomized controlled trials (RCTs), quasi-experimental studies, cohort studies, and case-control studies; and (4) outcomes: measures related to respiratory mechanics, ventilation parameters, patient-centered outcomes, and complication rates. Exclusion criteria included: (1) animal studies, reviews, or case reports; (2) studies with critical missing data that were unavailable from authors; and (3) non-Chinese/English publications.

### Data collection and quality assessment

Two investigators independently extracted data using predefined electronic forms. Information collected included: first author's surname, year of publication, geographical setting, sample size, patient baseline characteristics (e.g., age, sex, ARDS etiology, Berlin stage), ECCO_2_R technical parameters (e.g., blood flow rate, membrane surface area, anticoagulation protocol), ventilation details, and outcome measures. Study quality was assessed using appropriate tools: the Cochrane Risk of Bias Tool was used for RCTs to evaluate randomization, allocation concealment, blinding, handling of missing data, and outcome reporting bias (11); the Newcastle-Ottawa Scale (NOS) was applied to observational studies to assess selection, comparability, and outcome/exposure domains (12).

### Statistical analysis

Weighted mean difference (WMD) with 95% confidence intervals (CI) was computed for continuous outcomes. Proportions and corresponding 95% CIs were calculated for dichotomous outcomes. Due to anticipated clinical and methodological heterogeneity across studies, a random-effects model was employed for all meta-analyses (13). The certainty of the evidence for primary outcomes was evaluated using the GRADE framework (Grading of Recommendations, Assessment, Development, and Evaluations). Two review authors independently rated the evidence for each outcome based on risk of bias, inconsistency, indirectness, imprecision, and publication bias (14). Heterogeneity was evaluated using the *I*^2^ statistic and Cochran's Q test, with *I*^2^ ≥ 50% or a Q-test *P*-value < 0.10 indicating substantial heterogeneity (15, 16). Sensitivity analyses using the leave-one-out method were performed to assess the robustness of the results and the influence of individual studies on heterogeneity (17). To further investigate potential sources of heterogeneity, we performed univariate random-effects meta-regression for the primary outcomes of respiratory mechanics according to study design, mean age, and male proportion (18). Prespecified subgroup analyses were conducted based on study design, patient age, and sex. Publication bias was evaluated through visual inspection of funnel plot symmetry and quantified using Egger's and Begg's tests (19, 20). All statistical analyses were performed using STATA version 12.0 (StataCorp LP, College Station, TX, USA), with a two-sided *P*-value < 0.05 considered statistically significant.

## Results

### Literature search

**Figure 1:**
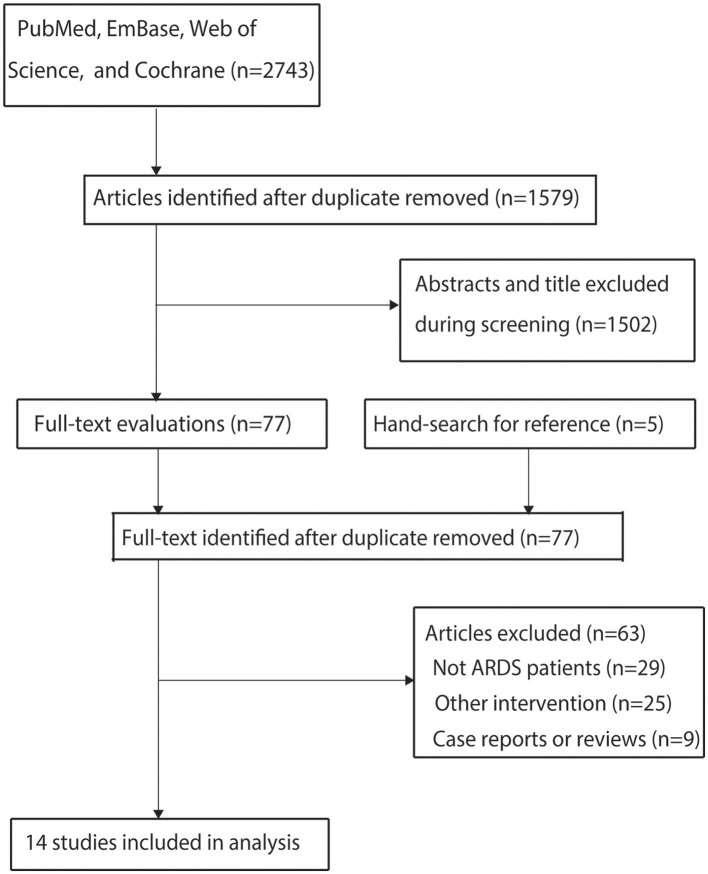
PRISMA flowchart illustrating the study selection process.

The initial electronic database search identified 2,743 articles. After removing duplicates, 1,579 studies remained. Screening of titles and abstracts excluded 1,502 studies as irrelevant to the research topic. The remaining 77 studies underwent full-text review, of which 63 were excluded for the following reasons: inclusion of non-ARDS patients (*n* = 29), absence of an ultra-protective ventilation strategy (*n* = 25), or study design as case reports or reviews (*n* = 9). A manual search of reference lists from relevant articles did not yield additional eligible studies. Ultimately, 14 studies met the inclusion criteria and were included in the meta-analysis (21–34). The literature search and study selection process are illustrated in Figure 1.

### Study characteristics

Table 1 summarizes the baseline characteristics of the included studies and patients. All studies were conducted in Europe and involved a total of 593 ARDS patients. Among them, two were RCTs, 10 were prospective observational studies, and two were retrospective observational studies. In recognition of the differing validity of evidence derived from various study designs, and in line with the reviewer's comment, we have stratified our primary analysis and interpretation based on this hierarchy. The inclusion of observational studies, including retrospective series, allows for the exploration of “real-world” feasibility and the spectrum of complications, but the estimates of physiological efficacy from these studies must be interpreted with caution due to inherent risks of confounding and selection bias. The results from the two RCTs are therefore emphasized as providing the most robust evidence within this dataset. The mean age of participants ranged from 49.8 to 70.0 years, and the proportion of male patients varied between 50.0% and 95.0%. Quality assessments of the included studies are provided in Supplementary File 2. The detailed risk of bias assessment is provided in Supplementary File 2. Of the two RCTs, one was judged to have a “low” risk of bias, while the other raised “some concerns” primarily due to unclear information regarding allocation concealment and blinding of participants and personnel. Observational studies were evaluated using the NOS, with three studies scoring 7 points, four scoring 6 points, and five scoring 5 points. The observational studies, as expected, were susceptible to selection bias and confounding due to their non-randomized design. This high risk of bias across the majority of the body of evidence is a key consideration when interpreting the pooled effect estimates, as it likely contributes to an overestimation of the treatment effects observed in the single-arm cohorts.

**Table 1:** Baseline characteristics of identified studies and involved patients.

**Study, year**	**Study design**	**Country**	**Sample size**	**Age (years)**	**Male (%)**	**PFR (mmHg)**	**PaCO_2_ (mmHg)**	**Disease status**
Terragni, 2009 (21)	Prospective observational	Italy	10	64.1	70.0	136.0	48.4	SAPS II: 56
Bein, 2013 (22)	RCT	Germany	40	49.8	95.0	152.0	57.3	Severe ARDS
Allardet-Servent, 2015 (23)	Prospective observational	France	11	70.0	73.0	133.0	48.0	SAPS II: 69
Fanlli, 2016 (24)	Prospective observational	Italy	15	55.0	73.0	159.0	51.0	SAPS II: 51
Peperstraete, 2017 (25)	Prospective pilot	Belgium	10	50.5	60.0	83.0	68.3	SAPS III: 72.5
Winiszewski, 2018 (26)	Retrospective chart review	France	33	63.0	60.6	145.0	50.3	IGS II: 49
Schmidt, 2018 (27)	Prospective pilot	France	20	60.0	55.0	188.0	43.0	SAPS II: 56
Augy, 2019 (28)	Prospective observational	France	70	66.0	71.0	131.0	58.0	SAPS II: 43
Combes, 2019 (29)	Prospective phase II	Italy	95	60.2	67.4	173.0	47.8	SAPS II: 45.9
Goursaud, 2021 (30)	Prospective observational	France	18	64.0	72.0	108.5	43.1	SAPS II: 42
McNamee, 2021 (31)	RCT	UK	202	60.2	68.0	153.1	55.8	APACHE II: 19
Chiumello, 2022 (32)	Prospective observational	Italy	10	65.0	50.0	115.0	49.0	Moderate to severe
Pasero, 2024 (33)	Retrospective case-control	Italy	14	65.0	64.3	121.5	60.5	SAPS II: 37
Monet, 2024 (34)	Prospective observational	France	45	60.0	71.0	128.0	46.0	SAPS II: 52

### Driving pressure, plateau pressure, and tidal volume

**Figure 2:**
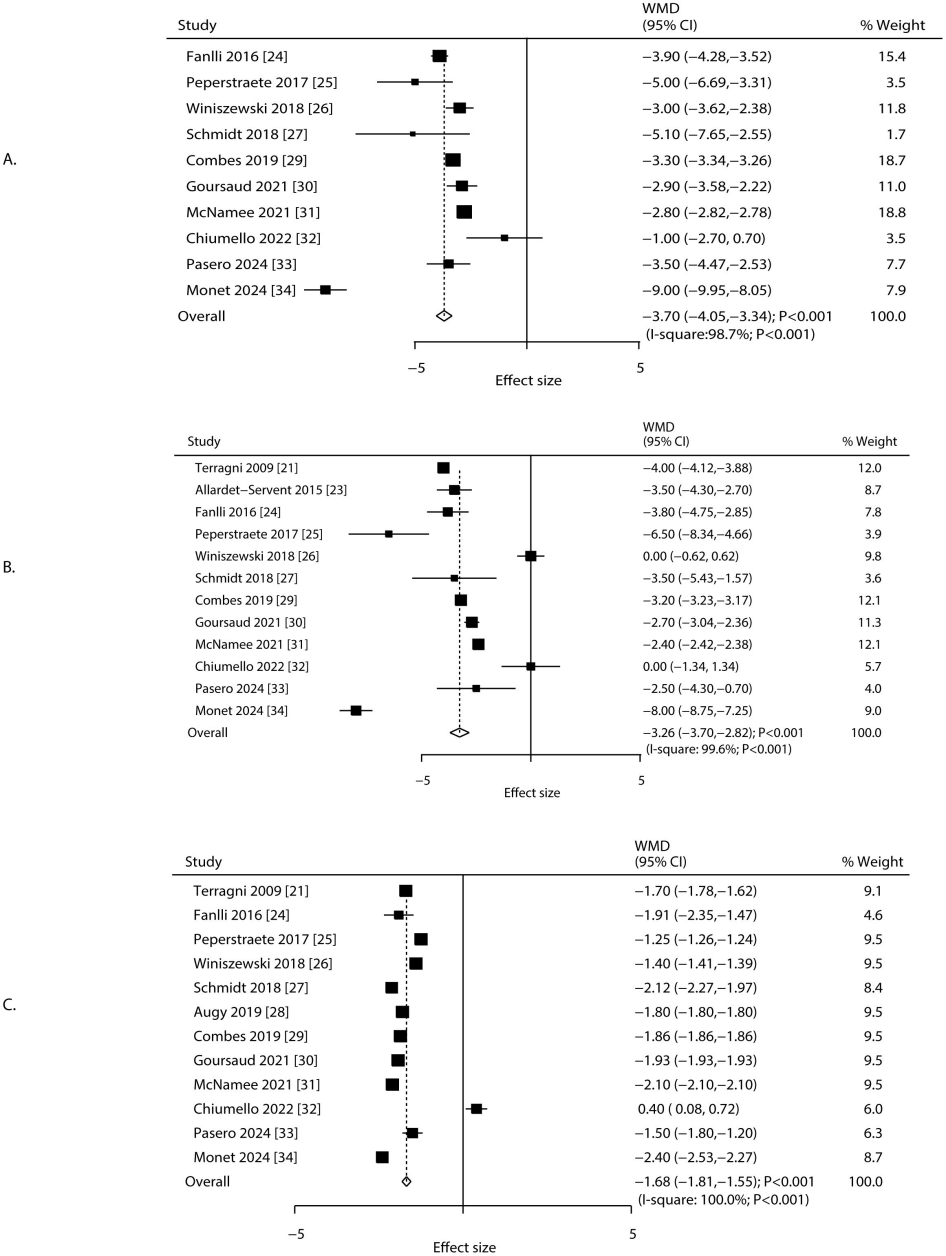
Changes in driving pressure, plateau pressure, and tidal volume after 24 h of ECCO_2_R, facilitating the application of ultraprotective ventilation. **(A)** Driving pressure, **(B)** plateau pressure, **(C)** tidal volume.

Changes in driving pressure, plateau pressure, and tidal volume after 24 h of ECCO_2_R-facilitated ultra-protective ventilation were reported in 10, 12, and 12 studies, respectively. Significant reductions were observed in driving pressure (WMD: −3.70 cm H_2_O; 95% CI: −4.05 to −3.34; *P* < 0.001), plateau pressure (WMD: −3.26 cm H_2_O; 95% CI: −3.70 to −2.82; *P* < 0.001), and tidal volume (WMD: −1.68 mL/kg; 95% CI: −1.81 to −1.55; *P* < 0.001; Figure 2). Considerable heterogeneity was detected for driving pressure (*I* ^2^ = 98.7%; *P* < 0.001), plateau pressure (*I*^2^ = 99.6%; *P* < 0.001), and tidal volume (*I*^2^ = 100.0%; *P* < 0.001). The consistently extreme heterogeneity (*I*^2^ > 95%) indicates that the included studies do not estimate a single common effect size, but rather a distribution of true effects. Therefore, the pooled WMD from the random-effects model should not be interpreted as a precise estimate of efficacy, but rather as a descriptive summary of the average reported effect across highly variable clinical settings, patient populations, and ECCO_2_R management protocols. The primary value of this analysis lies in confirming the direction of this effect and in exploring the potential sources of its substantial variability. Sensitivity analyses confirmed that the pooled estimates remained robust after sequential exclusion of individual studies (Supplementary File 3). Study design and male proportion were significant predictor affect the treatment effect of ECCO_2_R-facilitated ultra-protective ventilation on driving pressure, plateau pressure, and tidal volume, while mean age of patients was a significant predictor for tidal volume (Table 2). Subgroup analyses indicated significant reductions in driving pressure and tidal volume across all subgroups except among patients aged ≥65.0 years. Plateau pressure was significantly reduced in all subgroups except in retrospective studies and patients aged ≥65.0 years (Table 2). No significant publication bias was detected for driving pressure (Egger's *P* = 0.231; Begg's *P* = 0.858), plateau pressure (Egger's *P* = 0.422; Begg's *P* = 0.837), or tidal volume (Egger's *P* = 0.372; Begg's *P* = 0.945) at 24 h post-ECCO_2_R initiation (Supplementary File 4).

**Table 2:** Subgroup analyses for investigated outcomes.

**Outcomes**	**Factors**	**Subgroups**	**WMD and 95%CI**	***P*-value**	***I^2^* (%)**	**Q statistic**	**Meta-regression**
Driving pressure	Study design	Prospective observational	−4.31 (−5.42 to −3.19)	< 0.001	96.3	< 0.001	< 0.001
		Retrospective	−3.15 (−3.67 to −2.62)	< 0.001	0.0	0.395	
		RCT	−2.80 (−2.82 to −2.78)	< 0.001	–	–	
	Mean age (years)	≥65.0	−2.35 (−4.79 to 0.09)	0.059	84.0	0.012	0.965
		< 65.0	−3.82 (−4.19 to −3.44)	< 0.001	99.0	< 0.001	
	Male (%)	≥70.0	−5.24 (−8.03 to −2.45)	< 0.001	98.3	< 0.001	< 0.001
		< 70.0	−3.13 (−3.51 to −2.74)	< 0.001	98.8	< 0.001	
Plateau pressure	Study design	Prospective observational	−3.88 (−4.47 to −3.30)	< 0.001	97.8	< 0.001	< 0.001
		Retrospective	−1.10 (−3.53 to 1.33)	0.375	84.9	0.010	
		RCT	−2.40 (−2.42 to −2.38)	< 0.001	–	–	
	Mean age (years)	≥65.0	−2.03 (−4.31 to 0.26)	0.082	89.7	< 0.001	0.753
		< 65.0	−3.50 (−3.99 to −3.01)	< 0.001	99.7	< 0.001	
	Male (%)	≥70.0	−4.38 (−5.60 to −3.16)	< 0.001	97.6	< 0.001	< 0.001
		< 70.0	−2.25 (−2.81 to −1.68)	< 0.001	99.7	< 0.001	
Tidal volume	Study design	Prospective observational	−1.70 (−1.82 to −1.57)	< 0.001	100.0	< 0.001	< 0.001
		Retrospective	−1.40 (−1.41 to −1.39)	< 0.001	0.0	0.514	
		RCT	−2.10 (−2.10 to −2.10)	< 0.001	–	–	
	Mean age (years)	≥65.0	−0.97 (−2.19 to 0.25)	0.117	98.9	< 0.001	< 0.001
		< 65.0	−1.84 (−2.01 to −1.67)	< 0.001	100.0	< 0.001	
	Male (%)	≥70.0	−1.93 (−2.02 to −1.83)	< 0.001	99.8	< 0.001	< 0.001
		< 70.0	−1.45 (−1.70 to −1.20)	< 0.001	100.0	< 0.001	
PFR	Study design	Prospective observational	2.46 (−3.50 to 8.43)	0.418	0.0	0.997	0.004
		Retrospective	−1.77 (−68.31 to 64.77)	0.958	83.5	0.014	
		RCT	−5.30 (−8.99 to −1.61)	0.005	–	–	
	Mean age (years)	≥65.0	−14.29 (−45.54 to 16.96)	0.370	73.2	0.024	0.078
		< 65.0	−3.20 (−6.38 to −0.01)	0.049	0.0	0.462	
	Male (%)	≥70.0	6.25 (−3.83 to 16.33)	0.225	0.0	0.998	0.038
		< 70.0	−5.39 (−14.32 to 3.54)	0.237	56.7	0.031	
PEEP	Study design	Prospective observational	0.67 (−0.20 to 1.54)	0.130	98.5	< 0.001	< 0.001
		Retrospective	1.13 (0.69 to 1.58)	< 0.001	0.0	0.330	
		RCT	0.30 (0.29 to 0.31)	< 0.001	–	–	
	Mean age (years)	≥65.0	0.15 (−0.90 to 1.21)	0.775	93.8	< 0.001	< 0.001
		< 65.0	0.94 (0.71 to 1.16)	< 0.001	98.3	< 0.001	
	Male (%)	≥70.0	1.16 (−0.82 to 3.14)	0.251	99.1	< 0.001	< 0.001
		< 70.0	0.28 (0.16 to 0.39)	< 0.001	90.5	< 0.001	
Respiratory rate	Study design	Prospective observational	−0.57 (−2.77 to 1.62)	0.607	99.7	< 0.001	< 0.001
		Retrospective	−1.00 (−1.52 to −0.48)	< 0.001	–	–	
		RCT	2.70 (2.68 to 2.72)	< 0.001	–	–	
	Mean age (years)	≥65.0	0.00 (−0.21 to 0.21)	1.000	0.0	1.000	< 0.001
		< 65.0	−0.28 (−3.47 to 2.91)	0.865	100.0	< 0.001	
	Male (%)	≥70.0	0.40 (−1.29 to 2.08)	0.645	98.1	< 0.001	< 0.001
		< 70.0	−0.86 (−4.60 to 2.87)	0.650	100.0	< 0.001	

### PaO_2_/FiO_2_, positive end-expiratory pressure, and respiratory rate

**Figure 3:**
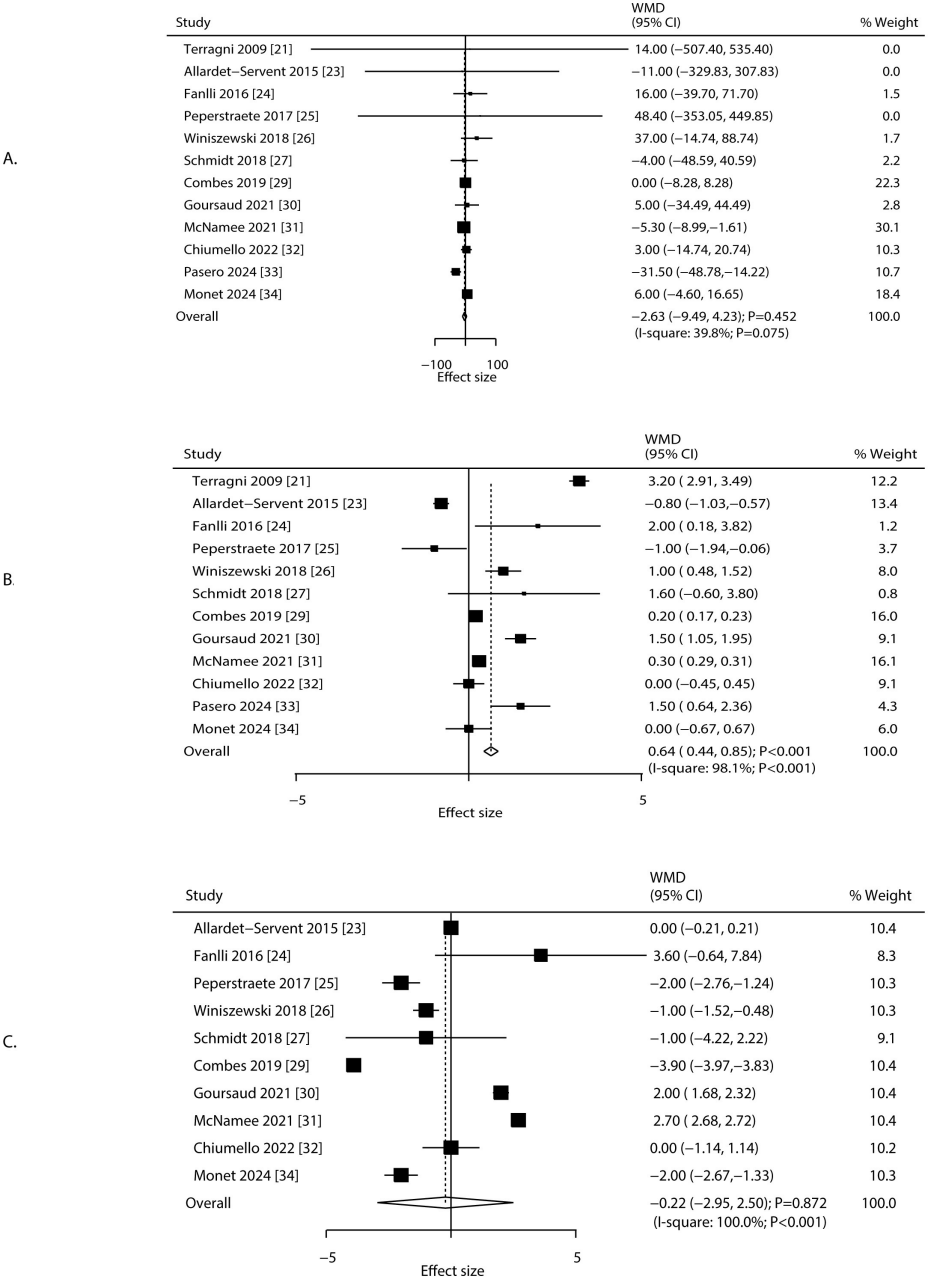
Changes in PaO_2_/FiO_2_, PEEP, and respiratory rate after 24 h of ECCO_2_R, facilitating the application of ultra-protective ventilation. **(A)** PFR, **(B)** PEEP, **(C)** respiratory rate.

Changes in PaO_2_/FiO_2_, positive end-expiratory pressure (PEEP), and respiratory rate after 24 h of ECCO_2_R were reported in 12, 12, and 10 studies, respectively (Figure 3). No statistically significant changes were observed in PaO_2_/FiO_2_ (WMD: −2.63; 95% CI: −9.49 to 4.23; *P* = 0.452) or respiratory rate (WMD: −0.22 breaths/min; 95% CI: −2.95 to 2.50; *P* = 0.872). In contrast, PEEP increased significantly (WMD: 0.64 cm H_2_O; 95% CI: 0.44 to 0.85; *P* < 0.001). Heterogeneity was significant for PaO_2_/FiO_2_ (*I*^2^ = 39.8%; *P* = 0.075), PEEP (*I*^2^ = 98.1%; *P* < 0.001), and respiratory rate (*I*^2^ = 100.0%; *P* < 0.001). Sensitivity analyses supported the stability of these pooled estimates (Supplementary File 3). The treatment effect of ECCO_2_R-facilitated ultra-protective ventilation on PaO_2_/FiO_2_ could affected by study design and male proportion, while study design, mean age, and male proportion were significant predictors for PEEP, and respiratory rate (Table 2). Subgroup analyses indicated that PaO_2_/FiO_2_ decreased significantly in RCTs and among patients aged < 65.0 years. PEEP increased significantly in most subgroups except in retrospective studies, patients aged ≥65.0 years, and studies with ≥70.0% male participants. Respiratory rate decreased significantly in retrospective studies but increased in RCTs (Table 2). No significant publication bias was identified for PaO_2_/FiO_2_ (Egger's *P* = 0.483; Begg's *P* = 0.631), PEEP (Egger's *P* = 0.539; Begg's *P* = 0.945), or respiratory rate (Egger's *P* = 0.310; Begg's *P* = 0.210) at 24 h after ECCO_2_R initiation (Supplementary File 4).

### pCO_2_ and pH

**Figure 4:**
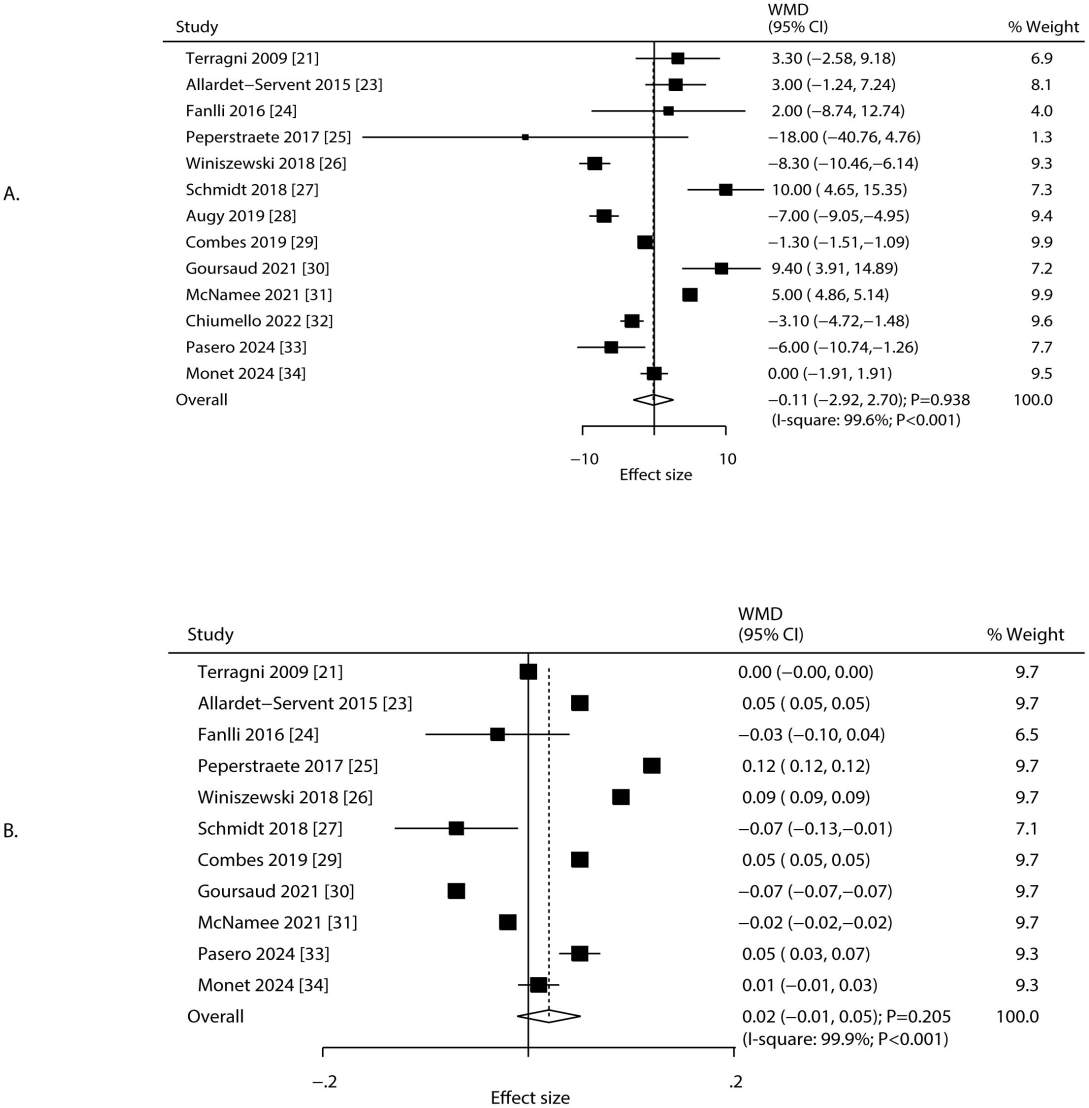
Changes in pCO_2_ and pH after 24 h of ECCO_2_R, facilitating the application of ultra-protective ventilation. **(A)** pCO_2_, **(B)** pH.

Changes in pCO_2_ and pH after 24 h of ECCO_2_R were reported in 13 and 11 studies, respectively (Figure 4). The implementation of ECCO_2_R-assisted ultra-protective ventilation resulted in no significant net change in pCO_2_ (WMD: −0.11 mmHg; 95% CI: −2.92 to 2.70; *P* = 0.938) or pH (WMD: 0.02; 95% CI: −0.01 to 0.05; *P* = 0.205) at 24 h. Substantial heterogeneity was observed for both pCO_2_ (*I*^2^ = 99.6%; *P* < 0.001) and pH (*I*^2^ = 99.9%; *P* < 0.001). Sensitivity analyses confirmed that the results were not driven by any single study (Supplementary File 3). The treatment effect of ECCO_2_R-facilitated ultra-protective ventilation on pCO_2_ and pH could affected by study design, mean age, and male proportion (Table 2). Subgroup analyses showed that pCO_2_ decreased significantly in retrospective studies but increased in RCTs. pH increased significantly in retrospective studies and among patients aged ≥65.0 years, but decreased in RCTs (Table 2). No significant publication bias was detected for pCO_2_ (*P*-value for Egger: 0.399; *P*-value for Begg: 0.360), or pH (*P*-value for Egger: 0.687; *P*-value for Begg: 0.638) at 24 h after initiating ECCO_2_R (Supplementary File 4).

### Mortality and complications

**Figure 5:**
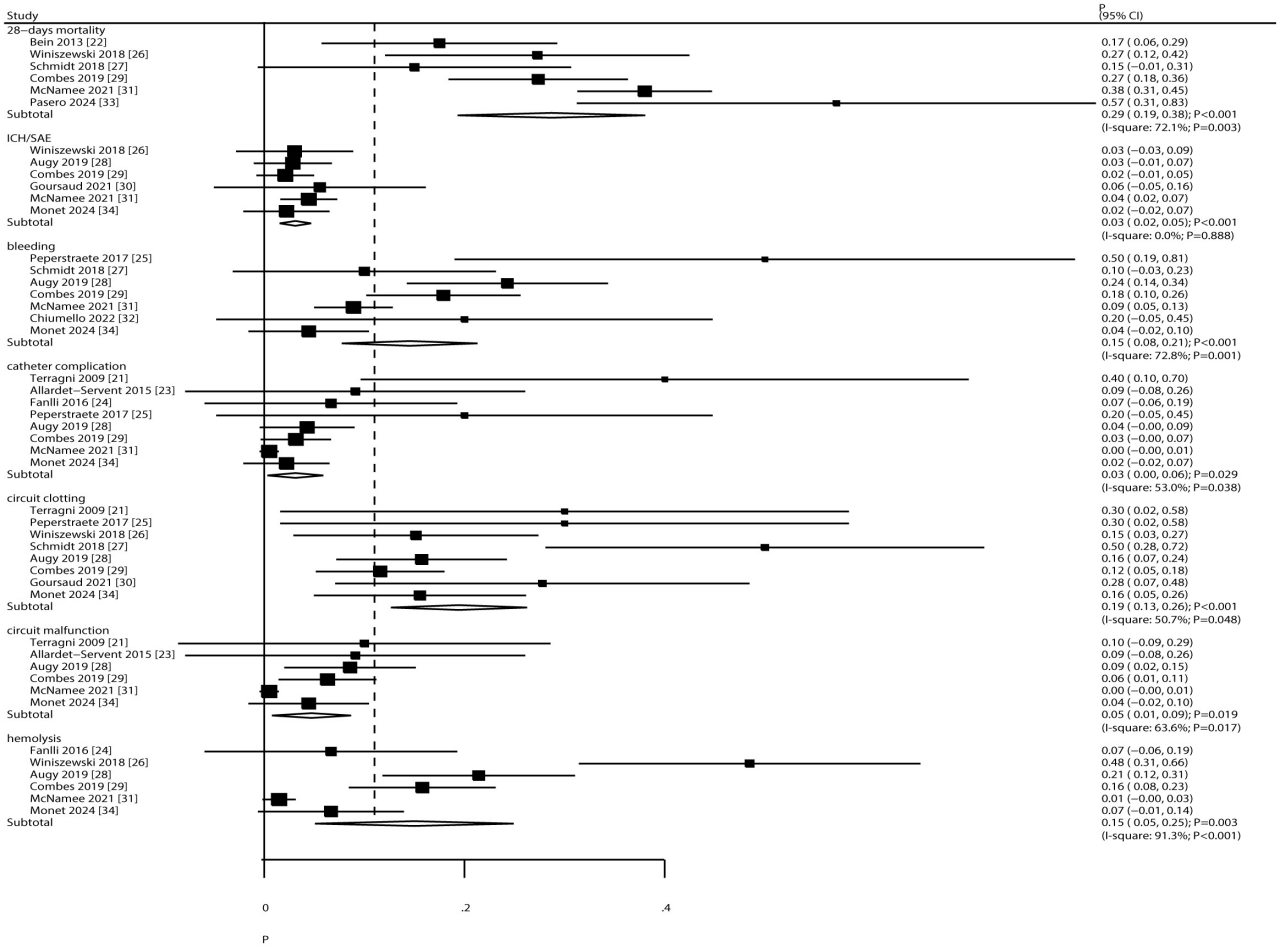
Pooled incidence of 28-day mortality and complications.

The pooled incidence of 28-day mortality and complications is presented in Figure 5. The overall 28-day mortality rate was 29% (95% CI: 19%−38%). The incidence of specific complications was as follows: intracranial hemorrhage/subarachnoid events (ICH/SAE), 3% (95% CI: 2%−5%); bleeding, 15% (95% CI: 8%−21%); catheter complications, 3% (95% CI: 0%−6%); circuit clotting, 19% (95% CI: 13%−26%); circuit malfunction, 5% (95% CI: 1%−9%); and hemolysis, 15% (95% CI: 5%−25%). Significant heterogeneity was observed for 28-day mortality (*I*^2^ = 72.1%; *P* = 0.003), bleeding (*I*^2^ = 72.8%; *P* = 0.001), catheter complications (*I*^2^ = 53.0%; *P* = 0.038), circuit clotting (*I*^2^ = 50.7%; *P* = 0.048), circuit malfunction (*I*^2^ = 63.6%; *P* = 0.017), and hemolysis (*I*^2^ = 91.3%; *P* < 0.001). No heterogeneity was observed for ICH/SAE (*I*^2^ = 0.0%; *P* = 0.888).

### Quality of evidence

The certainty of the evidence for the primary outcomes was formally evaluated using the GRADE framework. As summarized in Supplementary File 5, the overall certainty of evidence was rated as low for all outcomes. The primary reasons for downgrading the evidence were: (1) risk of bias, due to the predominance of non-randomized observational studies which are susceptible to confounding; and (2) inconsistency, due to substantial statistical heterogeneity (*I*^2^ > 90%) in the pooled estimates for most respiratory mechanics parameters. Consequently, while the point estimates suggest significant improvements in respiratory mechanics, the true magnitude of these effects is uncertain, and the findings should be interpreted with caution.

## Discussion

This meta-analysis demonstrates that ECCO_2_R-assisted ultra-protective ventilation facilitates clinically meaningful adjustments in ventilation parameters—consistent with lung-protective goals—without directly altering the inherent respiratory mechanics, which remained unchanged. Specifically, ECCO_2_R enables reductions in driving pressure (−3.70 cmH_2_O), plateau pressure (−3.26 cmH_2_O), and tidal volume (−1.68 mL/kg) by mitigating CO_2_ retention, thereby allowing the safe implementation of ultra-protective ventilation without the risk of severe hypercapnia. These adjustments are intended to reduce ventilator-induced lung injury but do not modify the underlying pathophysiology of ARDS. These findings help reconcile the conflict between lung protection and CO_2_ management inherent in conventional ventilation strategies. The observed increase in PEEP (0.64 cmH_2_O) suggests a synergistic effect with ECCO_2_R in promoting alveolar recruitment, which may further reduce ventilator-induced lung injury. This aligns with preclinical evidence indicating that low tidal volume combined with optimal PEEP decreases alveolar shear stress. However, the certainty of this evidence, as formally assessed by the GRADE framework, is low, primarily due to the inclusion of non-randomized studies with high risk of bias and substantial inconsistency. The effect sizes were consistently larger in observational studies than in the limited RCT data, underscoring the need for cautious interpretation.

Previous meta-analyses have evaluated the efficacy of ECCO_2_R. One synthesis of 49 studies confirmed that ECCO_2_R reduces PaCO_2_ and acidosis, facilitating less invasive ventilation. However, its impact on clinical outcomes remains uncertain, as RCTs showed no mortality benefit and reported more adverse events. That analysis did not specifically focus on ultra-protective strategies or exclude non-ARDS populations (35). Another meta-analysis of 10 studies found that ECCO_2_R-assisted ultra-protective ventilation reduced driving pressure by 3.56 cmH_2_O and tidal volume by 1.89 mL/kg from baseline, though no significant changes were observed in oxygenation, respiratory rate, or PEEP. Bleeding and hemolysis were frequently reported complications. However, the study was limited by incomplete literature retrieval and a lack of exploratory analyses (36). Our meta-analysis aimed to address these gaps by comprehensively evaluating the efficacy and safety of ECCO_2_R-facilitated ultra-protective ventilation specifically in patients with ARDS.

Our findings are consistent with previous reports indicating that ECCO_2_R helps maintain plateau pressure below 25 cmH_2_O. However, the reduction in driving pressure observed here (−3.70 cmH_2_O) exceeds the −1.2 cmH_2_O reported in earlier trials (37, 38). This discrepancy may stem from the inclusion of more observational studies, in which selection bias could amplify treatment effects. Although ECCO_2_R is primarily designed for CO_2_ removal, its neutral effects on PaO_2_/FiO_2_, pCO_2_, and pH are consistent with device mechanisms operating at low blood flow rates, which clear CO_2_ without improving oxygenation. The lack of a significant change in pCO_2_ and pH at 24 h should not be interpreted as ECCO_2_R having no effect on gas exchange. This finding is physiologically consistent with the intervention's purpose: ECCO_2_R was used to remove the additional CO_2_ produced by the deliberate reduction in minute ventilation. A stable pCO_2_ in the context of a significantly reduced tidal volume demonstrates the efficacy of ECCO_2_R in preventing severe hypercapnia, thereby “permitting” the use of ultra-protective settings (39). A true “control” group without ECCO_2_R would have been expected to develop significant respiratory acidosis. We have amended our abstract and conclusion to reflect this more precise interpretation.

The 28-day mortality rate of 29%—lower than that in conventional ARDS cohorts—should be interpreted cautiously due to the inclusion of only two RCTs and considerable heterogeneity (40). While ECCO_2_R facilitates improvements in respiratory mechanics, this meta-analysis highlights that its use is associated with a substantial and clinically important burden of device-related complications. The pooled incidence of bleeding was 15%, circuit clotting 19%, and hemolysis 15%. These are not trivial events; major bleeding can lead to hemodynamic instability and transfusion requirements, circuit clotting can abruptly terminate therapy and potentially cause thromboembolic events, and significant hemolysis can contribute to organ dysfunction (41). These complication rates critically inform the risk-benefit calculus for ECCO_2_R. The therapy's potential to reduce ventilator-induced lung injury must be weighed against its inherent risks of causing iatrogenic harm. The high rate of circuit clotting underscores the challenge of managing anticoagulation in a critically ill, often coagulopathic, population. Therefore, the application of ECCO_2_R should be reserved for settings with extensive expertise in extracorporeal support and vigilant monitoring for these specific complications. The “safety” of this approach is relative and is entirely dependent on a team's ability to prevent and manage these frequent adverse events.

The most significant finding of this meta-analysis may be the profound heterogeneity observed in the reported physiological effects of ECCO_2_R. Our meta-regression indicates that this variability is not random; it is partially explained by study design, mean age, and male proportion. Substantial heterogeneity (*I*^2^ >95%) was observed in driving and plateau pressures, attributable to three main factors: (1) variation in patient age (range: 49.8–70.0 years)—subgroup analysis showed attenuated respiratory improvements in patients aged ≥65 years, possibly due to comorbidities and reduced pulmonary compliance; (2) technical variations, including differences in blood flow rates, membrane surface areas, and anticoagulation protocols affecting CO_2_ clearance efficiency; and (3) inconsistent intervention strategies, such as strictly ultra-protective vs. transitional ventilation approaches. Prospective studies demonstrated greater reductions in plateau pressure compared to retrospective designs, underscoring the influence of study rigor. Additionally, the lack of a significant PEEP increase in subgroups with ≥70% male participants may suggest sex-related differences in chest wall compliance, warranting further investigation.

Based on these findings, early initiation of ECCO_2_R should be considered for patients with ARDS who exhibit a driving pressure >15 cmH_2_O during ventilation with a tidal volume of 6 mL/kg, to enable ultra-protective strategies. ECCO_2_R may also help stabilize transpulmonary pressure in patients with reduced chest wall compliance, such as those with obesity or thoracic deformities. Future high-quality RCTs should determine the optimal timing for ECCO_2_R initiation using 90-day mortality and ventilator-free days as primary outcomes. Research should also focus on developing predictive models that incorporate patient-specific factors and ECCO_2_R parameters to guide therapy titration. Technological advancements should aim to reduce dependence on systemic anticoagulation or implement regional anticoagulation strategies to minimize bleeding risks.

This study has several limitations: (1) inclusion of only two small-sample RCTs and a lack of large multicenter data; (2) absence of long-term outcomes such as ventilator-free days or organ recovery, limiting comprehensive prognostic assessment; (3) no direct parallel-group comparisons—future studies should compare ECCO_2_R-assisted ultra-protective ventilation directly with standard care in ARDS; (4) persistent heterogeneity despite subgroup and sensitivity analyses; and (5) reliance on published data, which may restrict in-depth exploration and increase the risk of publication bias.

## Conclusion

In conclusion, data from primarily observational studies suggest that ECCO_2_R is a feasible strategy to facilitate ultra-protective ventilation, leading to significant improvements in respiratory mechanics and preventing consequential hypercapnia. The certainty of this evidence is low, and the strategy is associated with a distinct profile of device-related risks. Therefore, it should not be considered a standard of care but rather a reserved intervention within a shared decision-making framework at experienced centers, pending further confirmation from high-quality RCTs.

## Data Availability

The original contributions presented in the study are included in the article/Supplementary material, further inquiries can be directed to the corresponding author.
