# Supplementary material for: Effects of extracorporeal carbon dioxide removal in facilitating ultra-protective ventilation strategies for patients with acute respiratory distress syndrome: a systematic review and meta-analysis
Source: Front Med (Lausanne). 2025 Nov 12;12:1707596. doi: 10.3389/fmed.2025.1707596 (PMC12648385; doi:10.3389/fmed.2025.1707596)
Supplement: Supplementary file 1 [file Table_1.docx]

**PubMed:**

#1 ("extracorporeal carbon dioxide removal" OR ECCO2R OR ECCO₂R OR "extracorporeal CO2 removal" OR "artificial lung" OR "pumpless lung assist")

#2 ("acute respiratory distress syndrome" OR ARDS OR "acute lung injury" OR ALI)

#3 ("ultraprotective ventilation" OR "ultra-protective ventilation" OR "very low tidal volume" OR "extreme lung protection")

#4 ("low tidal volume ventilation" OR LTVV OR "reduced tidal volume" OR "6 ml/kg" OR "4 ml/kg")

#5 #1 AND #2 AND (#3 OR #4)

#6 Filters: Humans

**Embase:**

#1 'extracorporeal carbon dioxide removal'/exp OR 'ecco2r'/exp OR 'artificial lung'/exp

#2 ('extracorporeal carbon dioxide removal' OR ECCO2R OR ECCO₂R OR 'extracorporeal CO2 removal' OR 'pumpless lung assist')

#3 #1 OR #2

#4 'acute respiratory distress syndrome'/exp OR 'adult respiratory distress syndrome'/exp

#5 ('acute respiratory distress syndrome' OR ARDS OR 'acute lung injury' OR ALI)

#6 #4 OR #5

#7 'protective ventilation'/exp OR 'tidal volume'/exp

#8 ('ultraprotective ventilation' OR 'ultra-protective ventilation' OR 'very low tidal volume' OR 'extreme lung protection')

#9 ('low tidal volume ventilation' OR LTVV OR 'reduced tidal volume' OR '6 ml/kg' OR '4 ml/kg')

#10 #7 OR #8 OR #9

#11 #3 AND #6 AND #10

#12 [humans]/lim AND [english]/lim)

**Web of Science:**

TS=(

("extracorporeal carbon dioxide removal" OR ECCO2R OR ECCO₂R OR "extracorporeal CO2 removal" OR "artificial lung")

AND

("acute respiratory distress syndrome" OR ARDS OR "acute lung injury")

AND

("ultraprotective ventilation" OR "ultra-protective ventilation" OR "very low tidal volume" OR "low tidal volume ventilation" OR "LTVV")

)

Refined by:

LANGUAGES: (ENGLISH)

**Cochrane Library:**

#1 [mh "Extracorporeal Circulation"] OR [mh "Carbon Dioxide"] OR [mh "Respiratory Therapy"]

#2 "extracorporeal carbon dioxide removal" OR ECCO2R OR ECCO₂R

#3 #1 OR #2

#4 [mh "Acute Respiratory Distress Syndrome"] OR [mh "Respiratory Distress Syndrome, Adult"]

#5 "acute respiratory distress syndrome" OR ARDS OR "acute lung injury"

#6 #4 OR #5

#7 [mh "Protective Ventilation"] OR [mh "Tidal Volume"]

#8 "ultraprotective ventilation" OR "ultra-protective ventilation" OR "very low tidal volume"

#9 "low tidal volume ventilation" OR LTVV OR "reduced tidal volume"

#10 #7 OR #8 OR #9

#11 #3 AND #6 AND #10 IN Cochrane Reviews, Trials, Clinical Answers
