# Supplementary material for: Effects of extracorporeal carbon dioxide removal in facilitating ultra-protective ventilation strategies for patients with acute respiratory distress syndrome: a systematic review and meta-analysis
Source: Front Med (Lausanne). 2025 Nov 12;12:1707596. doi: 10.3389/fmed.2025.1707596 (PMC12648385; doi:10.3389/fmed.2025.1707596)
Supplement: Supplementary file 2 [file Table_2.docx]

Table S1. The methodological quality of included RCTs

| Study | Random sequence generation | Allocation concealment | Blinding of participants and personnel | Blinding of outcome assessment | Incomplete outcome data | Selective reporting | Other bias |
| --- | --- | --- | --- | --- | --- | --- | --- |
| Bein 2013 [22] | Low | Unclear | Unclear | Low | Low | Low | Unclear |
| McNamee 2021 [31] | Low | Low | Low | Low | Low | Low | Unclear |

Table S2. The methodological quality of included observational studies

| Study | Selection | | | | Comparability | Outcome | | | NOS |
| --- | --- | --- | --- | --- | --- | --- | --- | --- | --- |
|  | Representativeness of the exposed cohort | Selection of the non exposed cohort | Ascertainment  of exposure | Demonstration that outcomes was not present at start of study | Comparability on the basis of the design or analysis | Assessment of outcome | Adequate follow-up duration | Adequate follow-up rate | Overall score |
| Terragni 2009 [21] | 0 | 0 | 1 | 1 | 1 | 1 | 0 | 1 | 5 |
| Allardet-Servent 2015 [23] | 0 | 0 | 1 | 1 | 1 | 1 | 1 | 1 | 6 |
| Fanlli 2016 [24] | 0 | 0 | 1 | 1 | 1 | 1 | 1 | 1 | 6 |
| Peperstraete 2017 [25] | 0 | 0 | 1 | 1 | 1 | 1 | 0 | 1 | 5 |
| Winiszewski 2018 [26] | 1 | 0 | 1 | 1 | 1 | 1 | 1 | 1 | 7 |
| Schmidt 2018 [27] | 0 | 0 | 1 | 1 | 1 | 1 | 1 | 1 | 6 |
| Augy 2019 [28] | 1 | 0 | 1 | 1 | 1 | 1 | 1 | 1 | 7 |
| Combes 2019 [29] | 1 | 0 | 1 | 1 | 1 | 1 | 1 | 1 | 7 |
| Goursaud 2021 [30] | 0 | 0 | 1 | 1 | 1 | 1 | 0 | 1 | 5 |
| Chiumello 2022 [32] | 0 | 0 | 1 | 1 | 1 | 1 | 0 | 1 | 5 |
| Pasero 2024 [33] | 0 | 0 | 1 | 1 | 1 | 1 | 0 | 1 | 5 |
| Monet 2024 [34] | 0 | 0 | 1 | 1 | 1 | 1 | 1 | 1 | 6 |
