# Supplementary material for: Effects of extracorporeal carbon dioxide removal in facilitating ultra-protective ventilation strategies for patients with acute respiratory distress syndrome: a systematic review and meta-analysis
Source: Front Med (Lausanne). 2025 Nov 12;12:1707596. doi: 10.3389/fmed.2025.1707596 (PMC12648385; doi:10.3389/fmed.2025.1707596)
Supplement: Supplementary file 3 [file Table_3.docx]

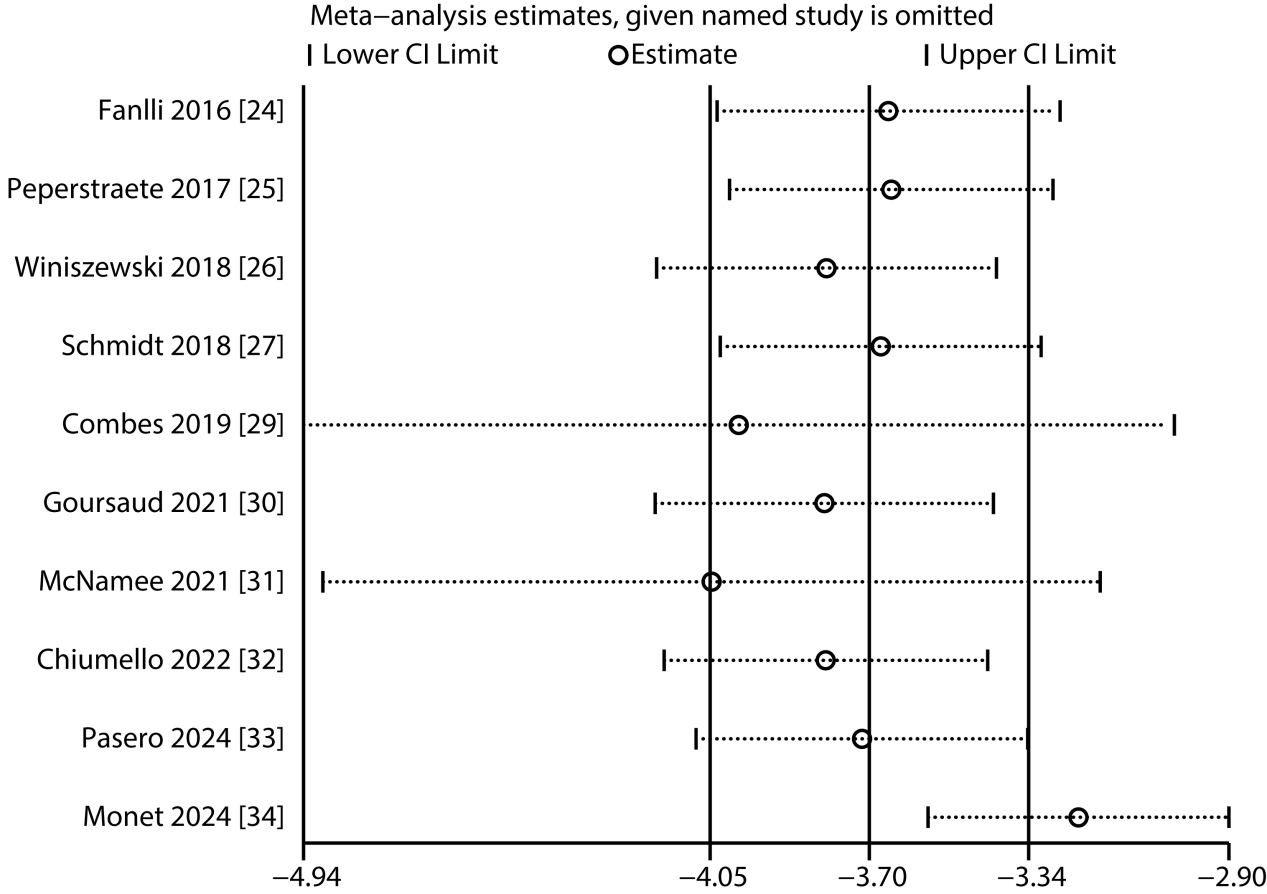


Figure S1. Sensitivity analysis for driving pressure


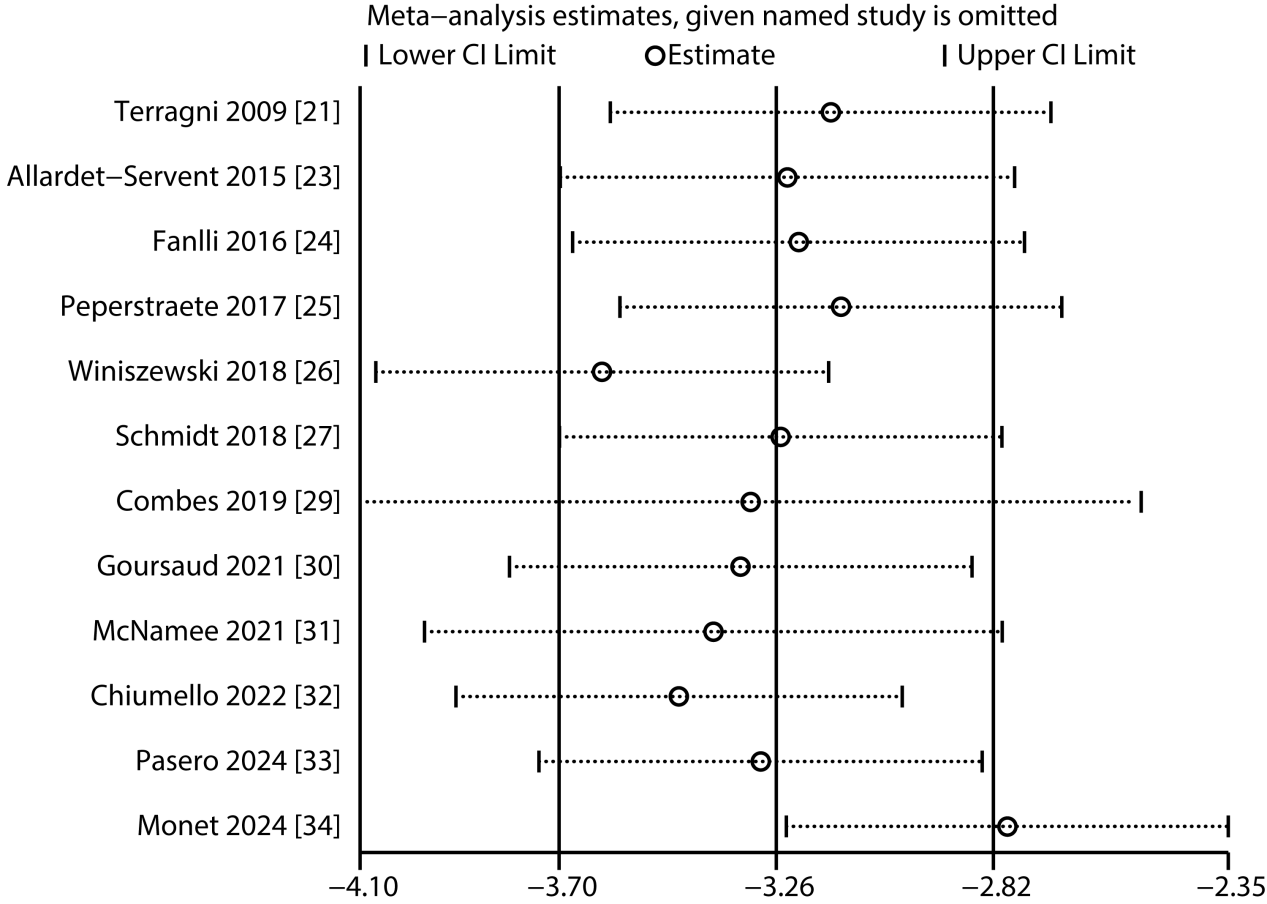


Figure S2. Sensitivity analysis for plateau pressure


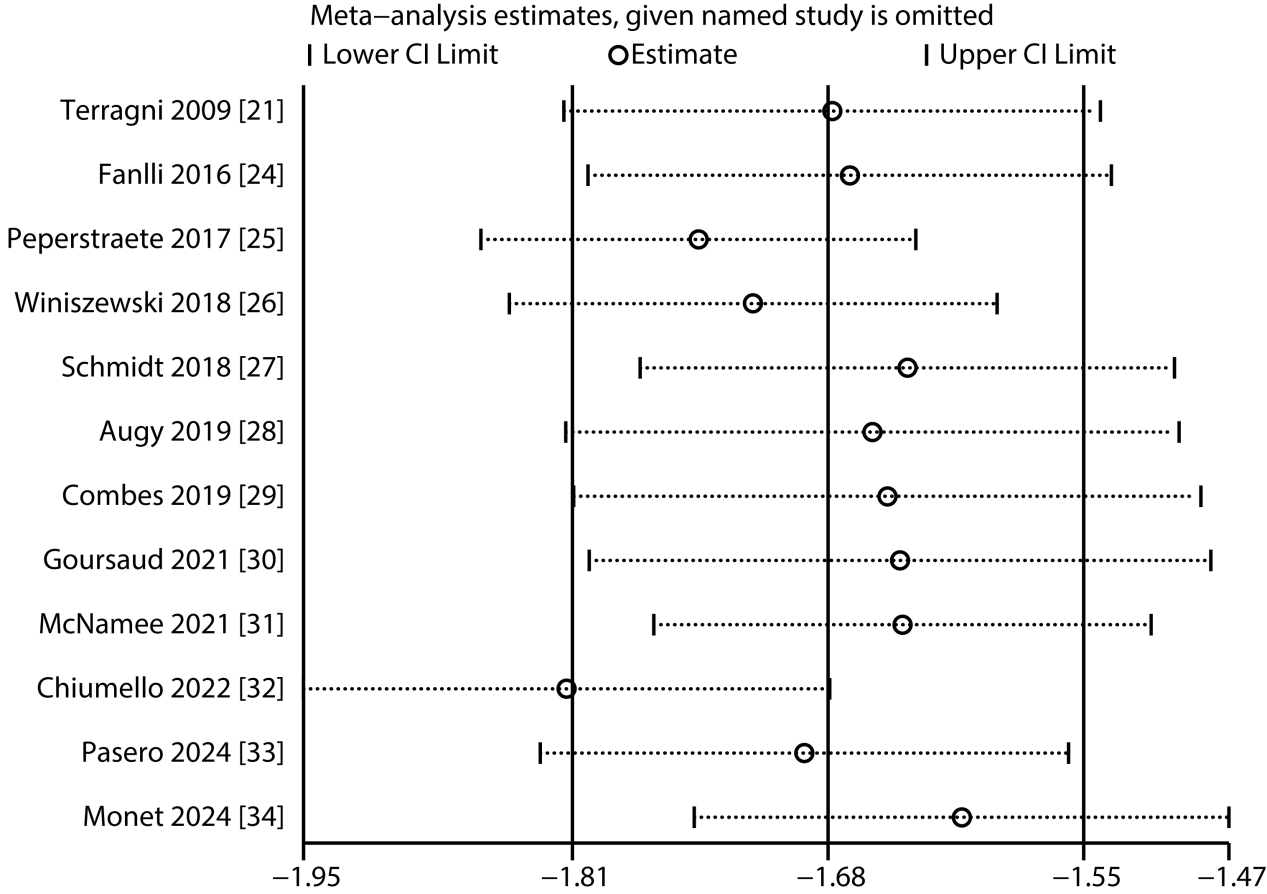


Figure S3. Sensitivity analysis for tidal volume


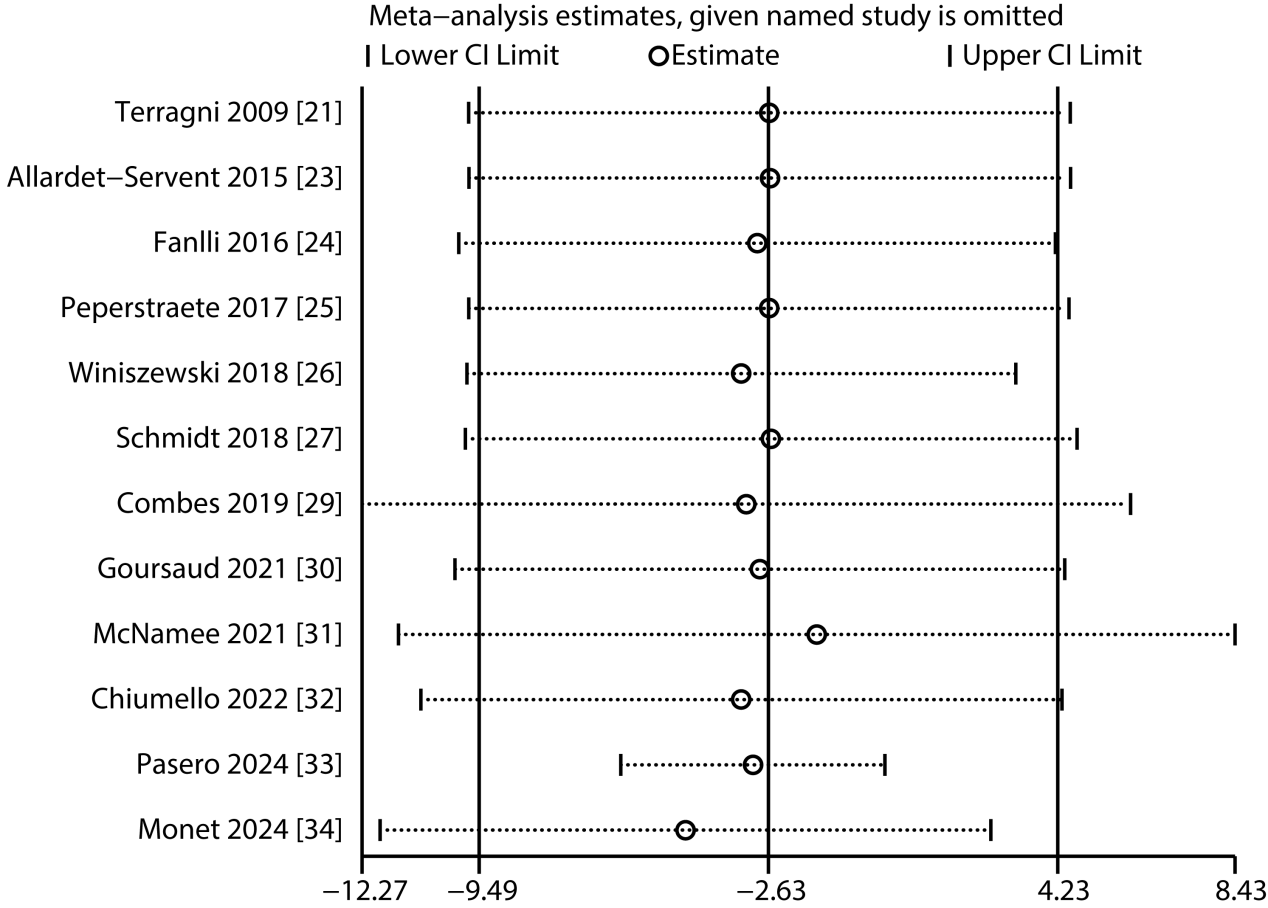


Figure S4. Sensitivity analysis for PFR


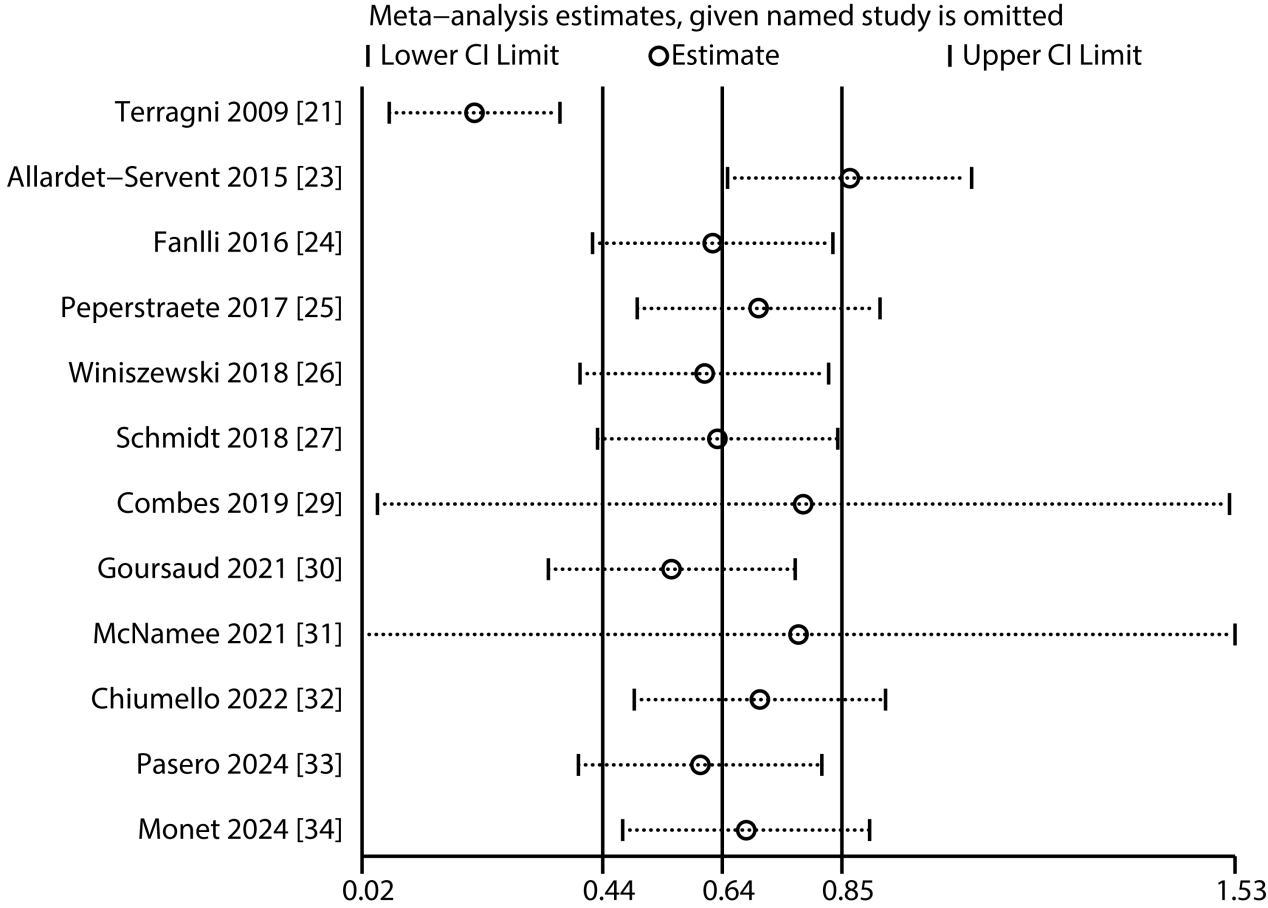


Figure S5. Sensitivity analysis for PEEP


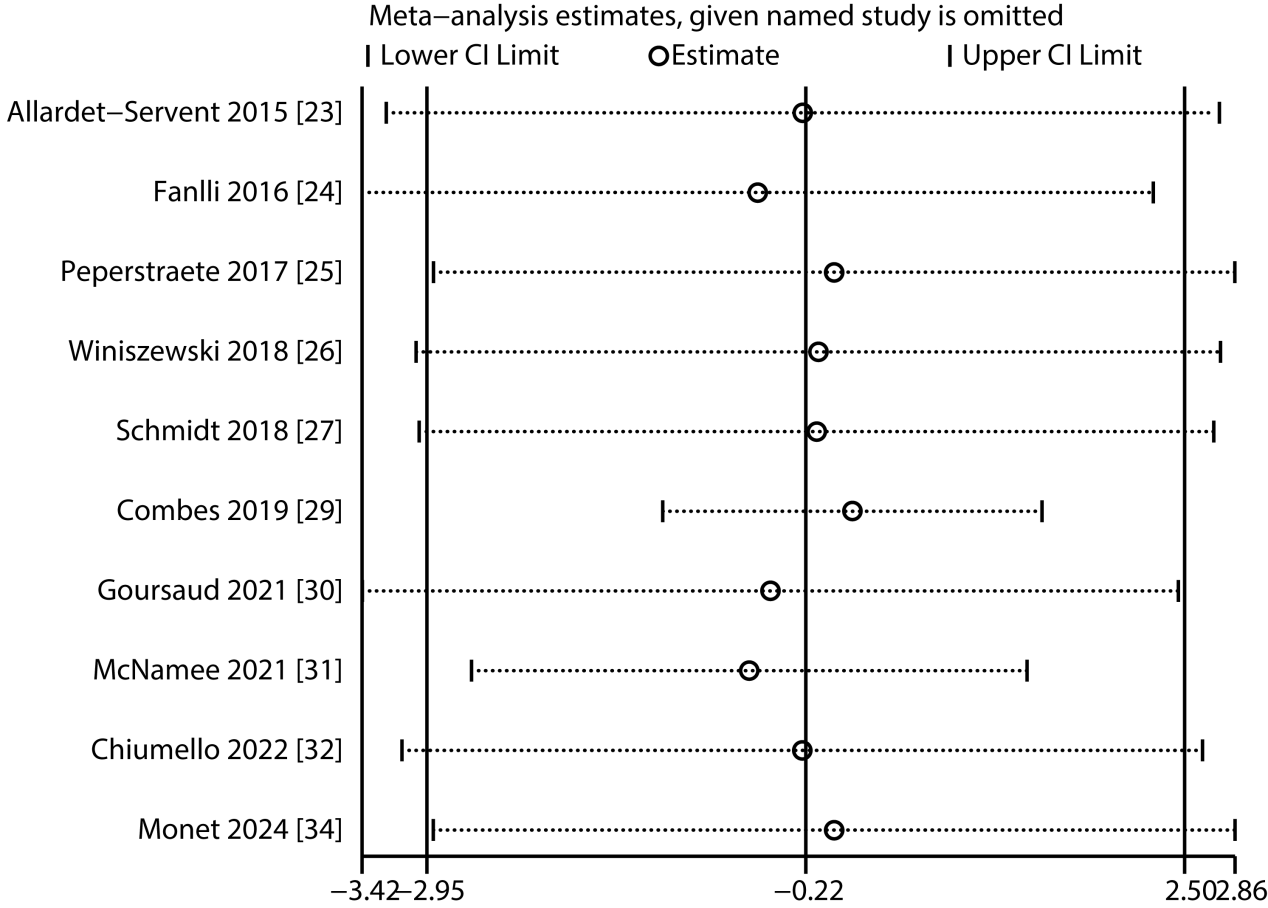


Figure S6. Sensitivity analysis for respiratory rate


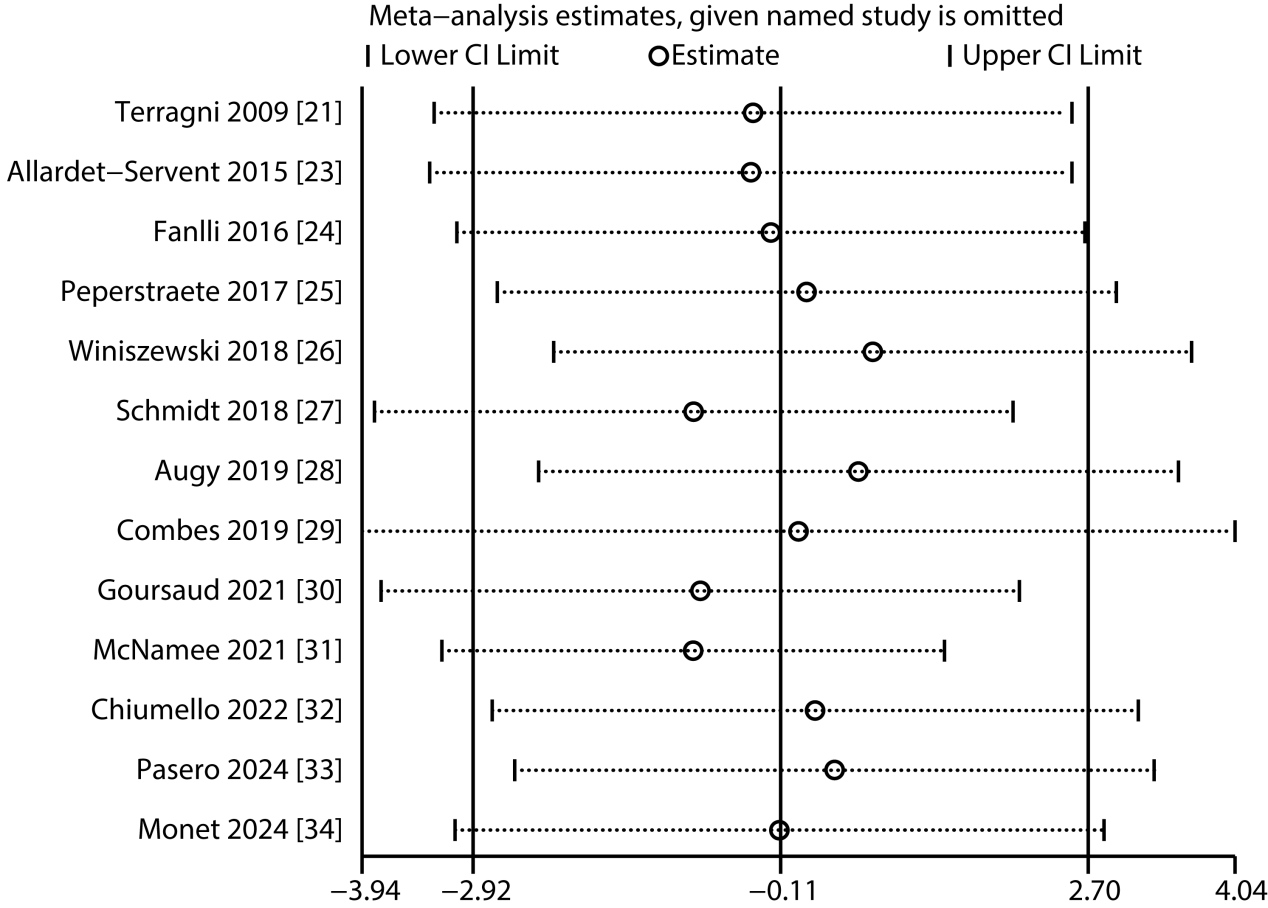


Figure S7. Sensitivity analysis for pCO_2_


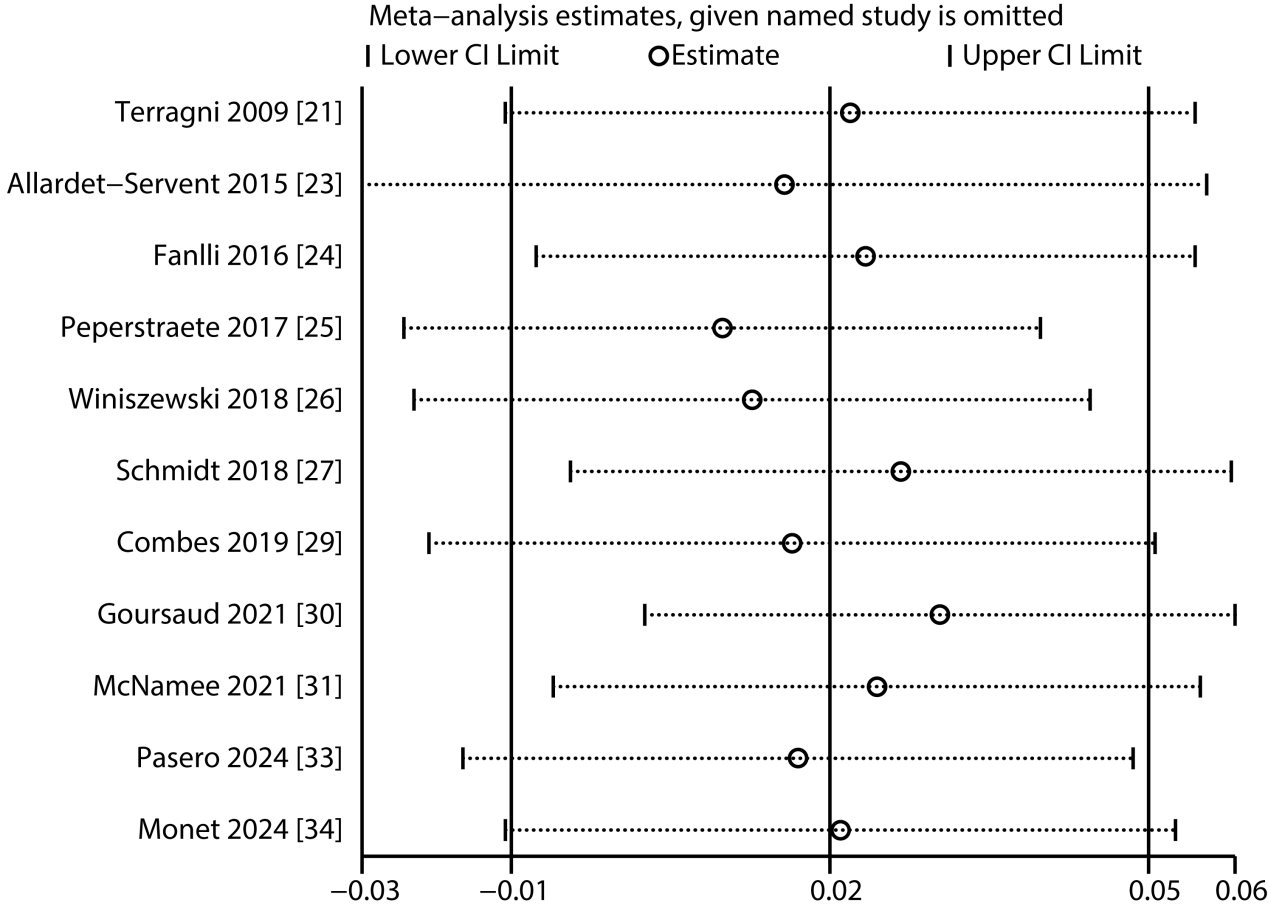


Figure S8. Sensitivity analysis for PH
