# Supplementary material for: Effects of extracorporeal carbon dioxide removal in facilitating ultra-protective ventilation strategies for patients with acute respiratory distress syndrome: a systematic review and meta-analysis
Source: Front Med (Lausanne). 2025 Nov 12;12:1707596. doi: 10.3389/fmed.2025.1707596 (PMC12648385; doi:10.3389/fmed.2025.1707596)
Supplement: Supplementary file 4 [file Table_4.docx]

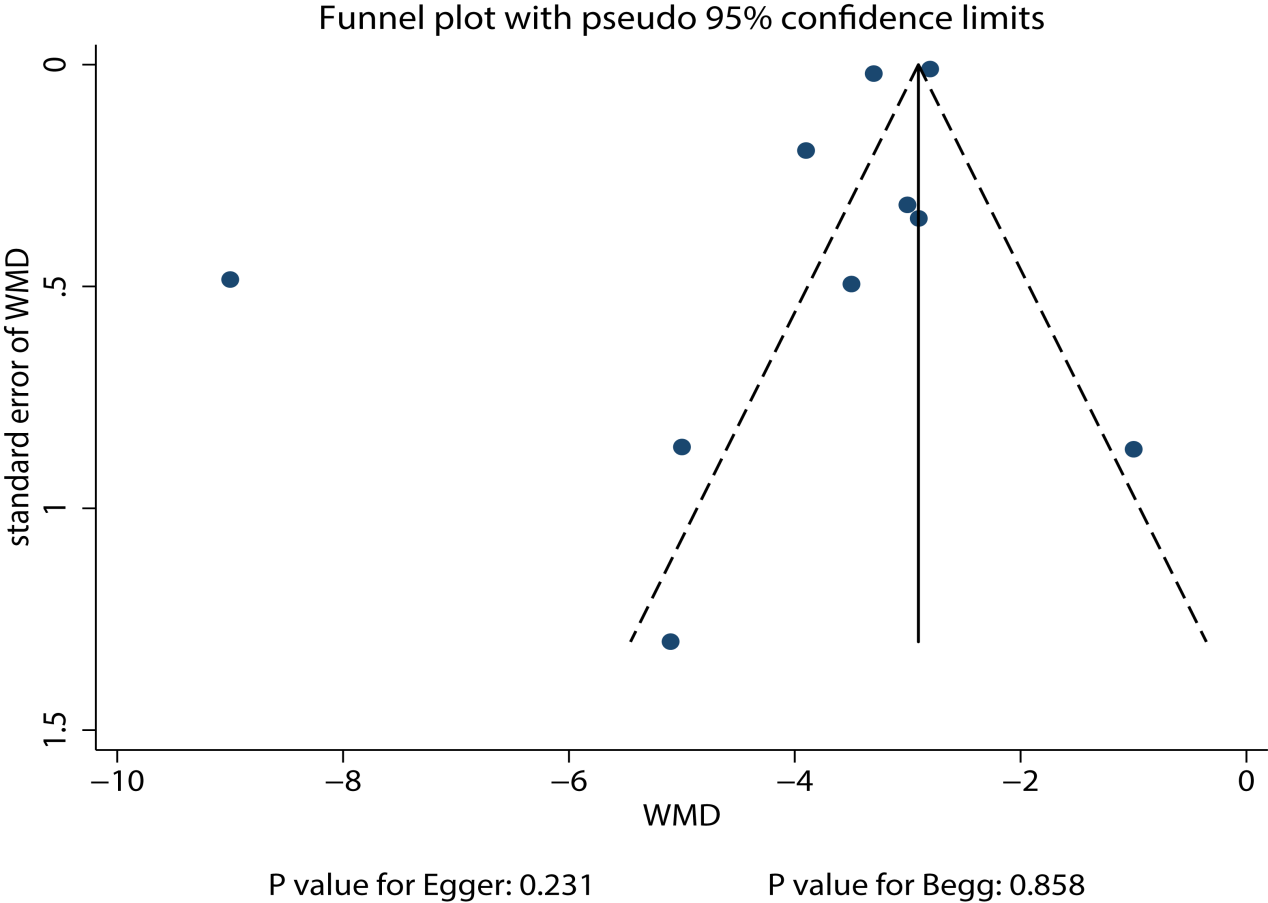


Figure S1. Funnel plot for driving pressure


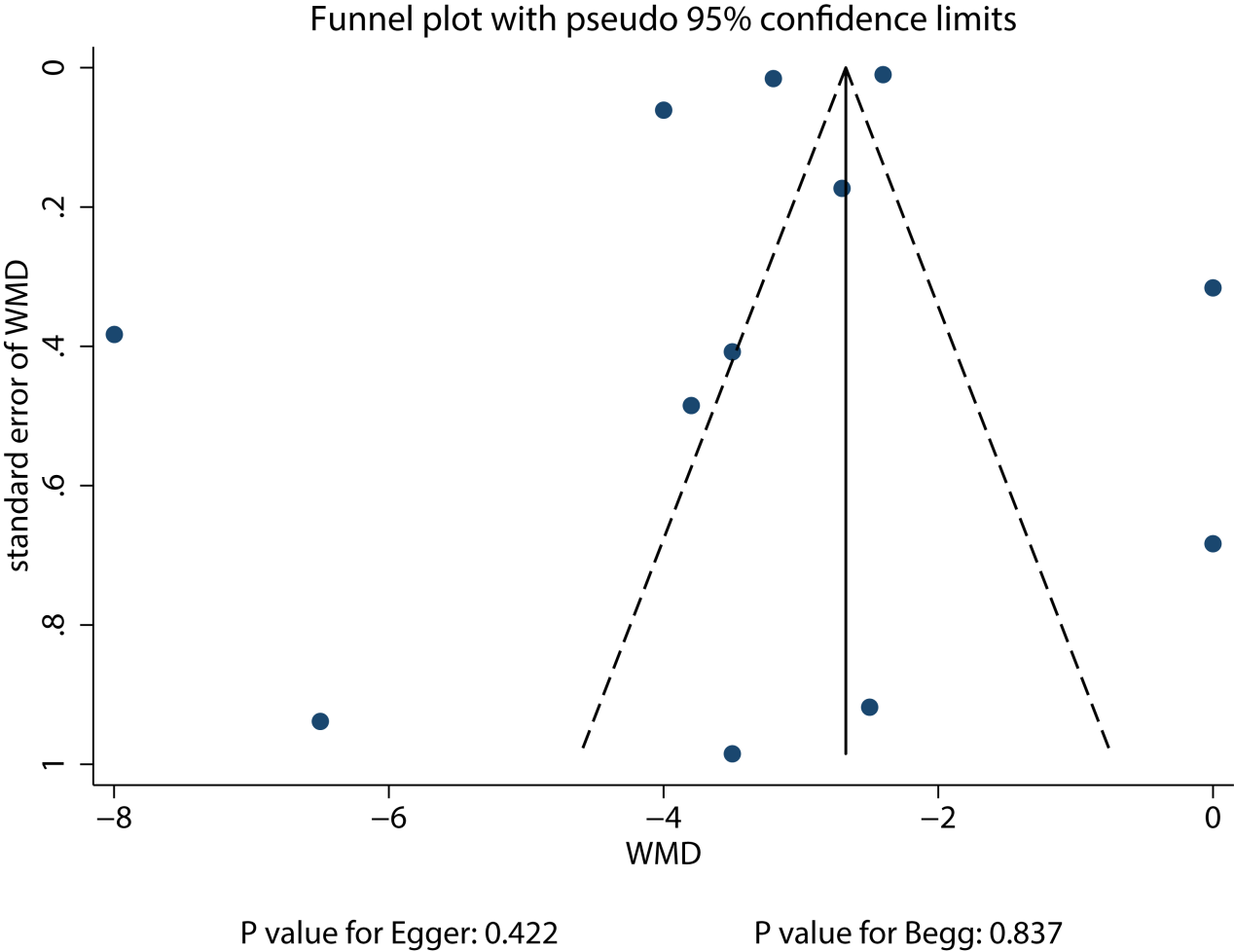


Figure S2. Funnel plot for plateau pressure


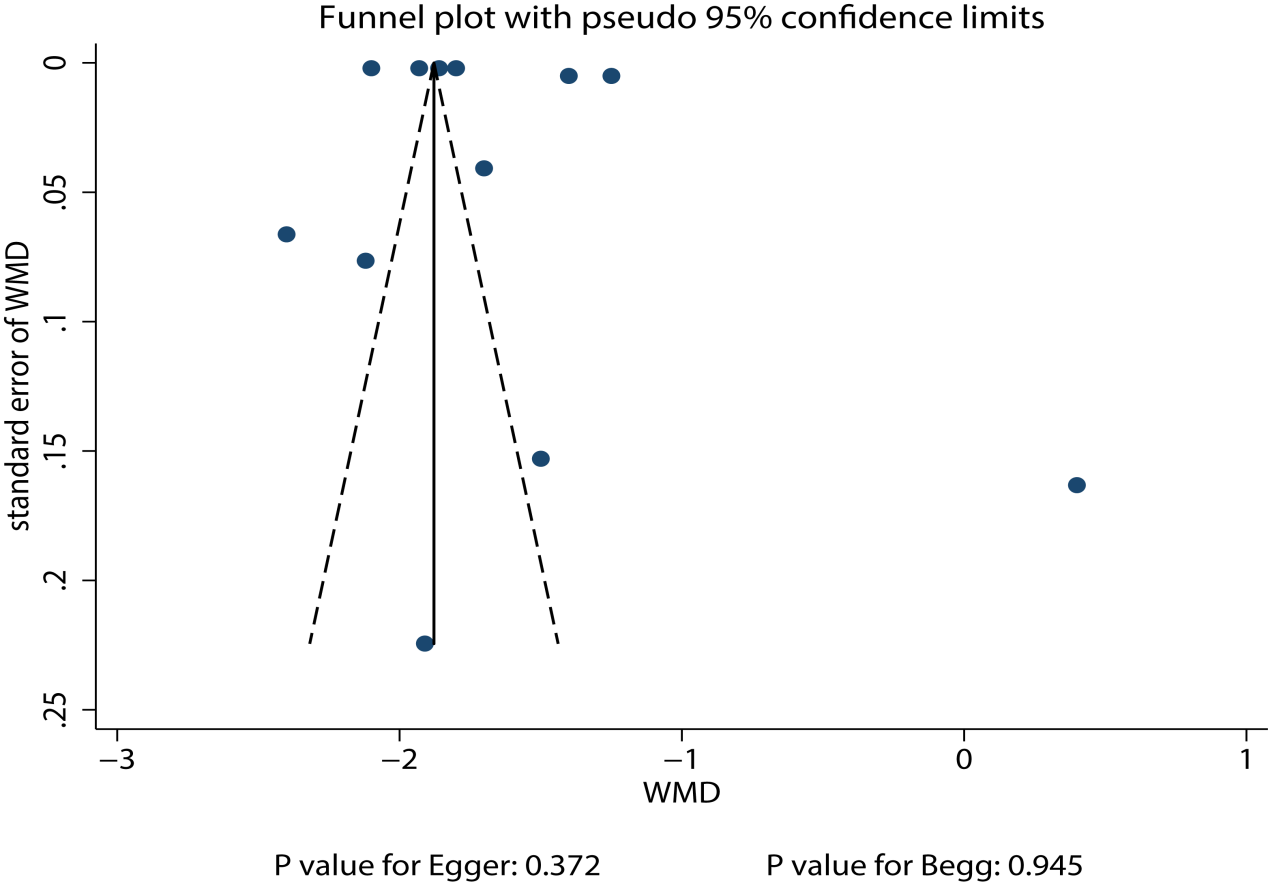


Figure S3. Funnel plot for tidal volume


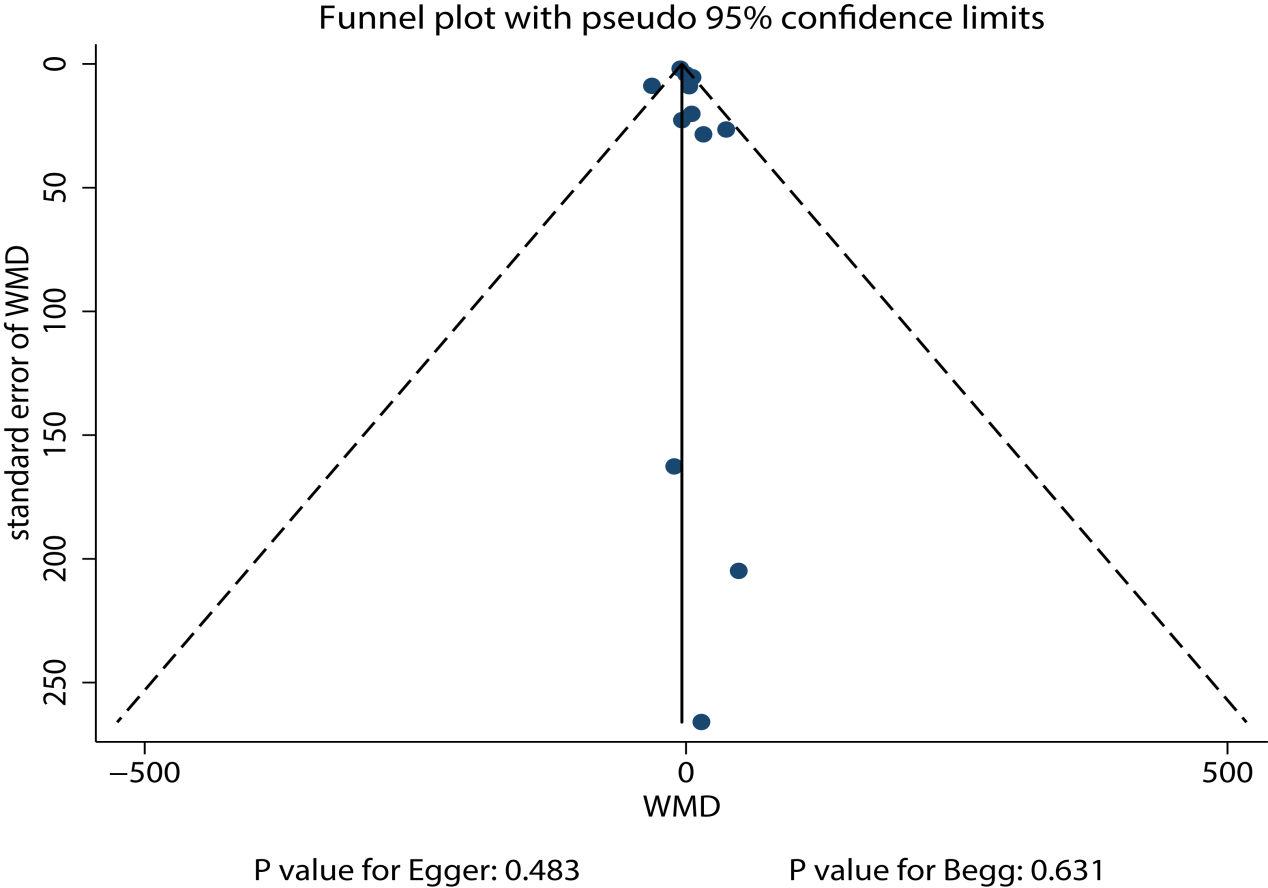


Figure S4. Funnel plot for PFR


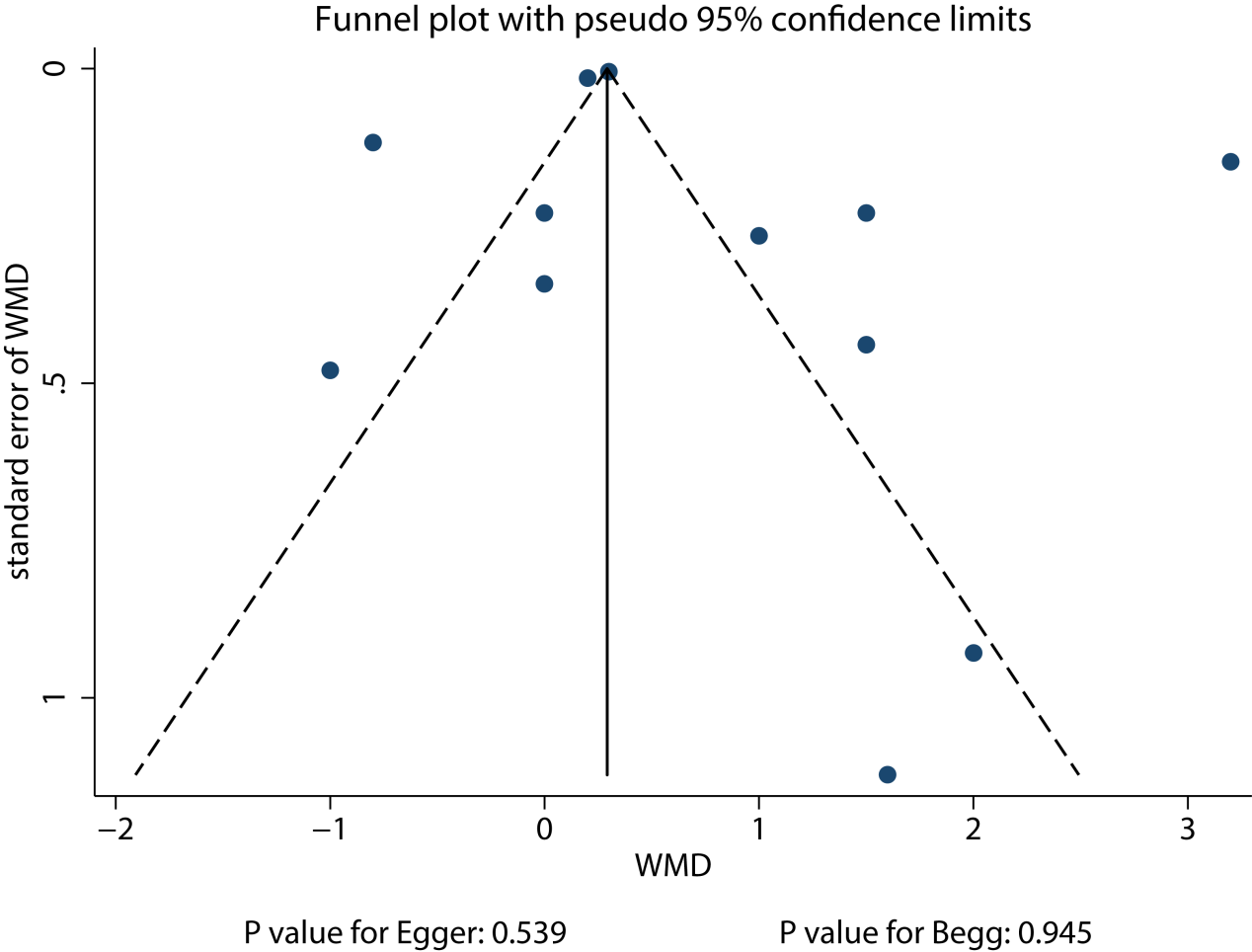


Figure S5. Funnel plot for PEEP


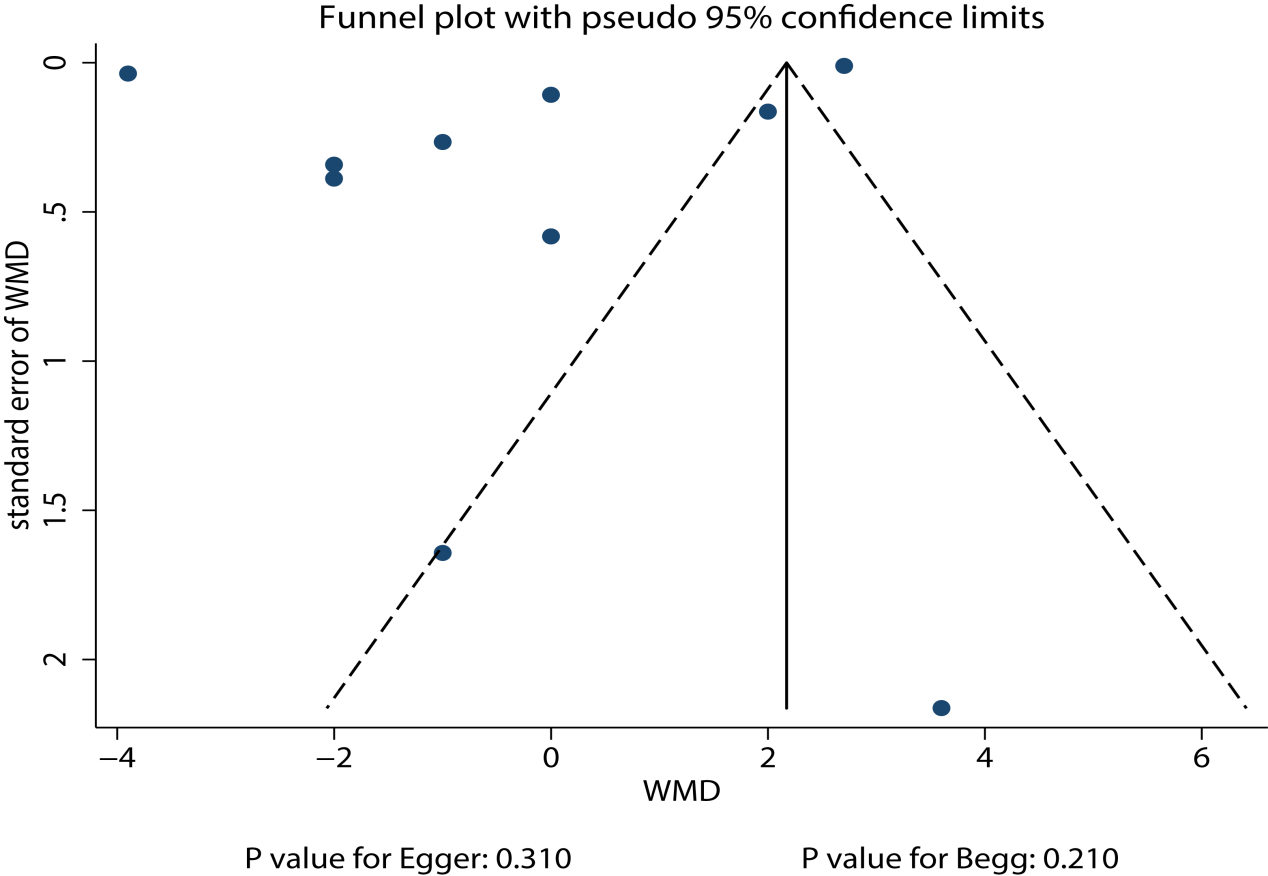


Figure S6. Funnel plot for respiratory rate


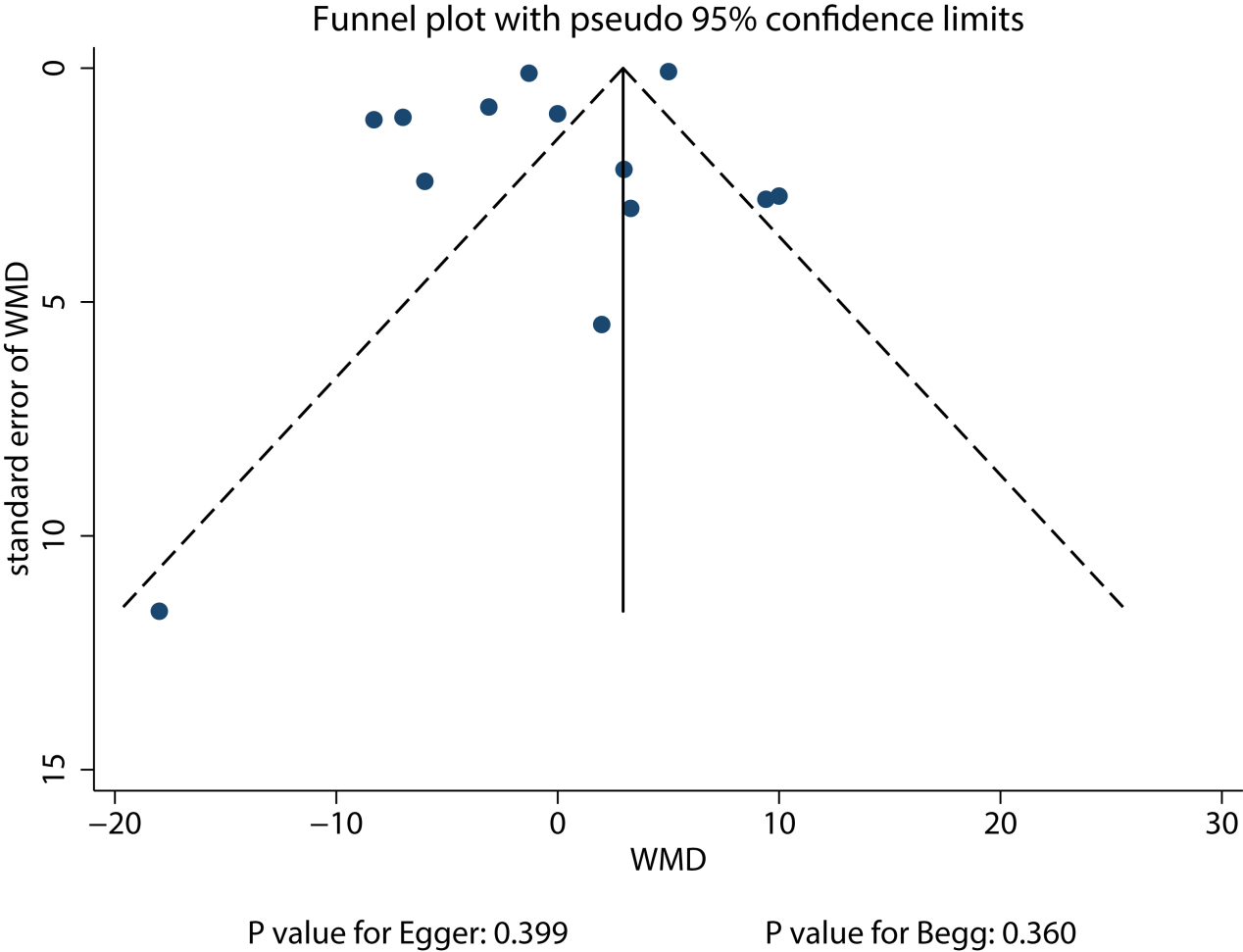


Figure S7. Funnel plot for pCO_2_


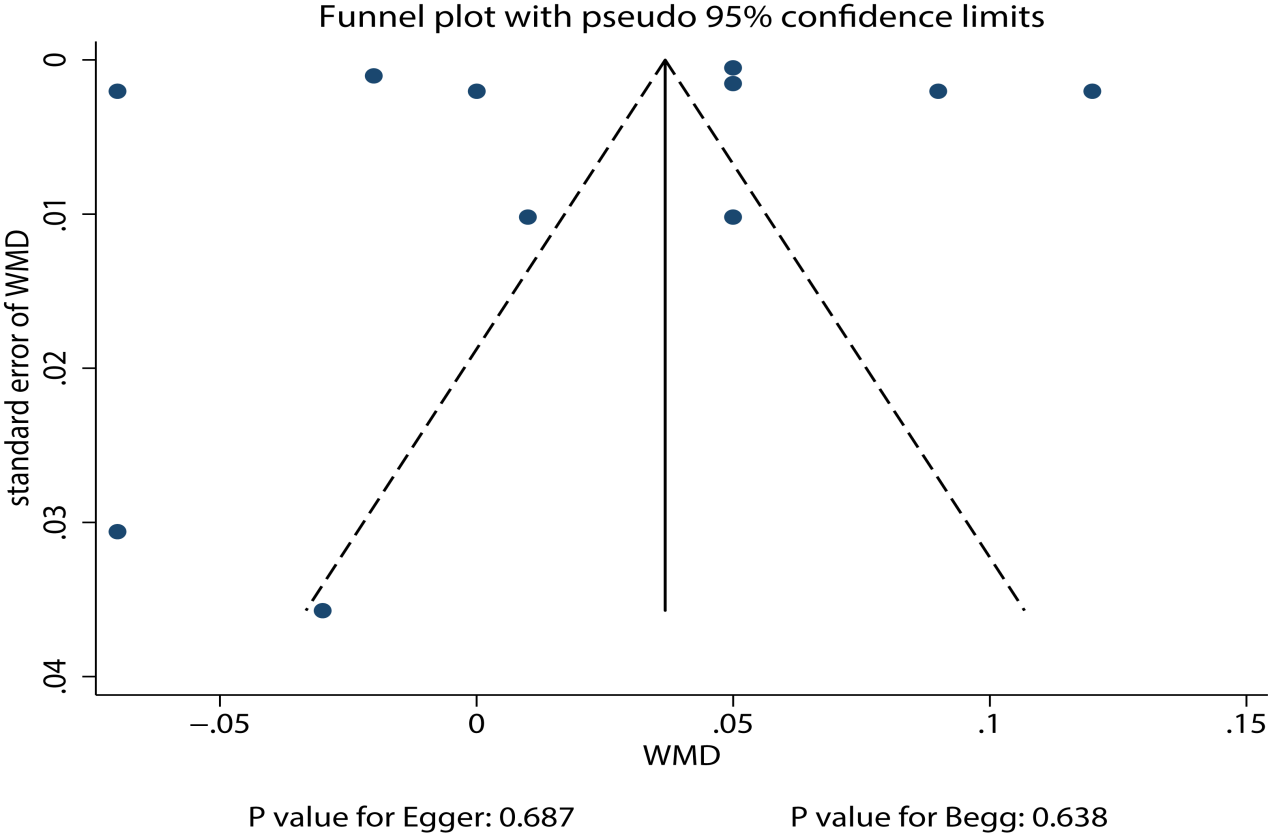


Figure S8. Funnel plot for PH
