# Supplementary material for: Effects of extracorporeal carbon dioxide removal in facilitating ultra-protective ventilation strategies for patients with acute respiratory distress syndrome: a systematic review and meta-analysis
Source: Front Med (Lausanne). 2025 Nov 12;12:1707596. doi: 10.3389/fmed.2025.1707596 (PMC12648385; doi:10.3389/fmed.2025.1707596)
Supplement: Supplementary file 5 [file Table_5.docx]

**Table S1: GRADE Evidence Profile for ECCO₂R-facilitated Ultra-Protective Ventilation in ARDS**

| Outcomes | Certainty of evidence | Comments | Reported Studies |
| --- | --- | --- | --- |
| Driving Pressure | Low | WMD: -3.70 (-4.05 to -3.34). A significant reduction was observed. | 1 RCT and 9 observational studies |
| Plateau Pressure | Low | WMD: -3.26 (-3.70 to -2.82). A significant reduction was observed. | 1 RCT and 11 observational studies |
| Tidal volume | Low | WMD: -1.68 (-1.81 to -1.55). A significant reduction was observed. | 1 RCT and 11 observational studies |
| PaO_2_/FiO_2_ | Low | WMD: -2.63 (-9.49 to 4.23). No significant change. | 1 RCT and 11 observational studies |
| Positive End-expiratory Pressure | Low | WMD: 0.64 (0.44 to 0.85). A significant elevation was observed. | 1 RCT and 11 observational studies |
| Respiratory Rate | Low | WMD: -0.22 (-2.95 to 2.50). No significant change. | 1 RCT and 9 observational studies |
| pCO_2_ | Low | WMD: -0.11 (-2.92 to 2.70). No significant change. | 1 RCT and 12 observational studies |
| pH | Low | WMD: 0.02 (-0.01 to 0.05). No significant change. | 1 RCT and 10 observational studies |
